# Transcriptomic Features of T Cell-Barren Tumors Are Conserved Across Diverse Tumor Types

**DOI:** 10.3389/fimmu.2020.00057

**Published:** 2020-02-13

**Authors:** Eric D. Routh, Ashok K. Pullikuth, Guangxu Jin, Julia Chifman, Jeff W. Chou, Ralph B. D'Agostino, Ken-ichiro Seino, Haruka Wada, Cristin G. Print, Wei Zhang, Yong Lu, Lance D. Miller

**Affiliations:** ^1^Department of Cancer Biology, Wake Forest School of Medicine, Winston-Salem, NC, United States; ^2^Wake Forest Baptist Comprehensive Cancer Center, Winston-Salem, NC, United States; ^3^Department of Mathematics and Statistics, American University, Washington, DC, United States; ^4^Department of Biostatistical Sciences, Wake Forest School of Medicine, Winston-Salem, NC, United States; ^5^Department of Immunobiology, Hokkaido University, Sapporo, Japan; ^6^Department of Molecular Medicine and Pathology, University of Auckland, Auckland, New Zealand; ^7^Maurice Wilkins Centre for Molecular Biodiscovery, University of Auckland, Auckland, New Zealand; ^8^Department of Microbiology and Immunology, Wake Forest School of Medicine, Winston-Salem, NC, United States

**Keywords:** tumor biology, immune evasion, tumor-infiltrating T cells, transcriptomics, bioinformatics, bone morphogenetic protein 7 (BMP7), REST corepressor 2 (RCOR2)

## Abstract

**Background:** Understanding how tumors subvert immune destruction is essential to the development of cancer immunotherapies. New evidence suggests that tumors limit anti-tumor immunity by exploiting transcriptional programs that regulate intratumoral trafficking and accumulation of effector cells. Here, we investigated the gene expression profiles that distinguish immunologically “cold” and “hot” tumors across diverse tumor types.

**Methods:** RNAseq profiles of tumors (*n* = 8,920) representing 23 solid tumor types were analyzed using immune gene signatures that quantify CD8+ T cell abundance. Genes and pathways associated with a low CD8+ T cell infiltration profile (CD8-Low) were identified by correlation, differential expression, and statistical ranking methods. Gene subsets were evaluated in immunotherapy treatment cohorts and functionally characterized in cell lines and mouse tumor models.

**Results:** Among different cancer types, we observed highly significant overlap of genes enriched in CD8-Low tumors, which included known immunomodulatory genes (e.g., BMP7, CMTM4, KDM5B, RCOR2) and exhibited significant associations with Wnt signaling, neurogenesis, cell-cell junctions, lipid biosynthesis, epidermal development, and cancer-testis antigens. Analysis of mutually exclusive gene clusters demonstrated that different transcriptional programs may converge on the T cell-cold phenotype as well as predict for response and survival of patients to Nivo treatment. Furthermore, we confirmed that a top-ranking candidate belonging to the TGF-β superfamily, BMP7, negatively regulates CD8+ T cell abundance in immunocompetent murine tumor models, with and without anti-PD-L1 treatment.

**Conclusions:** This study presents the first evidence that solid tumors of diverse anatomical origin acquire conserved transcriptional alterations that may be operative in the T cell-cold state. Our findings demonstrate the potential clinical utility of CD8-Low tumor-associated genes for predicting patient immunotherapy outcomes and point to novel mechanisms with potential for broad therapeutic exploitation.

## Introduction

The immune system plays a pivotal role in limiting cancer growth (1), and insights into the mechanisms that govern how immune cells sense, interface with, and respond to cancer have led to the development of immunotherapeutic strategies that enhance anti-tumor immunity. Critical to the establishment of effective anti-tumor immunity is the tumor-localized recruitment of antigen-specific CD8+ T cells and their subsequent activation, intratumoral migration, and resilience to immunosuppressive signals in the tumor microenvironment (TME). The abundance of tumor-infiltrating lymphocytes (TILs), and CD8+ T cells, in particular, is associated with favorable clinical prognoses in a wide range of solid malignancies, including melanoma and cancers of the head and neck, breast, bladder, ovaries, and colon (2). T cell infiltration associated with good prognosis is often accompanied by the presence of other activated proinflammatory cells [e.g., T_H_1 T cells, natural killer (NK) cells, and antigen-presenting cells (APCs)] indicative of a T cell-inflamed phenotype that has also been associated with a positive response to immune checkpoint blockade (ICB) (3–6). Moreover, an increase in CD8+ T cell tumor infiltration induced by ICB is associated with durable treatment response in both patients and animal models (7–9). Thus, the establishment and maintenance of an *immunologically hot* TME, characterized by abundant effector T cell infiltration, is clinically desirable.

By contrast, a non-T cell-inflamed or *immunologically cold* tumor state is associated with poor patient prognosis (6) and ICB non-responsiveness (10), and is believed to arise from mechanisms of immune suppression and evasion employed by cancer cells to avoid immune destruction (11). Mechanisms of tumor immune escape include antigen deletion, downregulation of antigen-presentation machinery, and the establishment of an immunosuppressive TME via PD-L1 upregulation or tumor cooption of immunosuppressive myeloid cells and regulatory T cells (12). Physical exclusion of CD8+ T cells by tumor enrichment of fibrotic stroma has also been associated with an immune-cold TME (13). However, the extent to which these mechanisms explain the immunologically cold phenotype of solid tumors is unclear. A number of studies indicate that the expression of certain TME- and tumor-derived factors can functionally limit the infiltration of CD8+ T cells into tumors, thereby attenuating anti-tumor immune responses. For example, VEGF, endothelin-1 (ET-1), and EGFL7 are tumor-secreted proteins that decrease cellular adhesion molecule (CAM) expression by tumor endothelium, which in turn blocks T cell transendothelial migration and subsequent trafficking of T cells into tumors (11, 14, 15). Pharmacological neutralization of the ET-1-endothelin B receptor (ET_B_R) signaling axis in a preclinical ovarian cancer model resulted in increased intratumoral CD8+ T cell infiltration and subsequent tumor response to an otherwise ineffective autologous cancer cell vaccine (14). In line with this and similar observations, the inability of CD8+ T cells to penetrate tumors is increasingly recognized as a contributing factor in immunotherapy failure (16). Thus, strategies to increase tumor penetration by CD8+ T cells via targeting mechanisms that restrict their intratumoral trafficking and accumulation would likely favor anti-tumor immunity and bolster the efficacy of current ICB therapies.

In the current work, we hypothesized that a comprehensive transcriptomic analysis of immunologically cold tumors would reveal candidate genes and pathways that may potentiate the negative regulation of effector cell abundance. Using an informatics-guided approach, we recently developed a *de novo* discovery platform for identifying immunological gene signatures from the TME that are conserved across solid tumors of diverse tissue origin (17). Composed of genes with immune-specialized functions, these gene signatures reflect the relative abundance of distinct tumor-infiltrating immune cell populations. Several T cell-related signatures that were identified showed strong correlation with previously reported gene signatures of effector cell subsets associated with reduced risk of distant metastasis, improved patient survival, and response to immunotherapy (17–23). Here, we utilize a CD8+ T cell-focused gene signature, quantified from RNAseq gene expression data, to investigate the relationship between intratumoral T cell abundance and tumor expression profiles in 23 solid tumor types. Using correlative and statistical ranking methods, we identify genes consistently overexpressed in CD8+ T cell-Low (CD8-Low) tumors, termed *candidate protein regulators of immune trafficking* (CulPRITs), and investigate their underlying biological properties. In the case of one such CulPRIT, bone morphogenetic protein 7 (BMP7), we demonstrate a functional role in limiting intratumoral CD8+ T cell abundance in murine tumor models.

## Materials and Methods

### Cancer Data Sets and Metagene Construction

The Cancer Genome Atlas (TCGA) solid tumor data sets composed of 100 or more tumor samples were accessed from Firebrowse.org (Broad Institute, MIT/Harvard). Level 3 Illumina HiSeq RNAseqV2 data (RSEM-normalized) was downloaded and log_2_-transformed (with pseudocount +1). Tumor data sets were culled to exclude non-cancer tissue specimens. Metagene scores were calculated for each tumor by taking the geometric mean of the log_2_ expression values of (1) CD8A, CD8B, CD3D, and CD3E (T^SIG^); or (2) GZMA, GZMB, GNLY, and PRF1 (C^SIG^). Relative to Figure 7, the Riaz et al. RNAseq data set of melanoma biopsies pre- or on-nivolumab (Nivo) treatment (24) was accessed via the Gene Expression Omnibus (accession no. GSE91061). FPKM normalized data in the form originally processed by Riaz et al. was used in our analyses. Supplementary Table 2 of that publication was used to align patient clinical characteristics with corresponding RNAseq profiles. Patient 3 RNAseq data were omitted according to the authors' recommendations. In total, the 96 samples from patients annotated for tumor responses by RECIST v1.1 criteria were utilized [pre-treatment (1–7 days prior to first dose), *n* = 48; on-treatment (days 23–29), *n* = 48]. Genes comprising the metagene signatures were mapped to this data set using the NHGRI's HGNC Multi-Symbol Checker (https://www.genenames.org/tools/multi-symbol-checker/). Signature scores were computed as the geometric mean of the log_2_ expression values. Signature quartiles were established in pre- and on-treatment samples, independently. Gene identifiers comprising the C1 and C2 signatures derived from the median percentile rank (MPR) CulPRITs (Figure 4B), their corresponding metagene scores, and associated clinical annotations are shown in Supplementary Datasheet 1. Of note, the C1 and C2 signatures derived from the exact binomial probability (EBP) CulPRITs (Figure 6B) were also analyzed in the Riaz et al. data set and found to possess similar or lesser therapy-predictive and prognostic associations (data not shown).

### Gene Ranking Metrics

For each TCGA tumor group, immune signature scores were used to rank genes for their association with the CD8+ T cell-cold state. Two ranking strategies were employed. In the first, tumors were partitioned into tertiles based on immune signature (T^SIG^ or C^SIG^) scores, and differential gene expression analysis was performed comparing tumor profiles of the low vs. high tertiles using the “Limma” R package (25). For each gene (*n* = 20,501), the average log fold change (LFC method) and FDR-corrected *q* values were computed. Genes were ranked on LFC, where negative values reflected genes overexpressed in CD8-Low tumors relative to CD8-High tumors, and then assigned a corresponding percentile rank (0–100), with higher percentile ranks corresponding to genes more highly expressed in CD8-Low tumors. In the second strategy, Spearman correlation analysis (SC method) was applied to assess the correlation between tumor immune signature scores and tumor gene expression profiles. Similar to above, genes were ranked and assigned percentile ranks using the Spearman correlation coefficient, where the most negatively correlated genes were assigned a higher relative percentile rank. To analyze the significance of genes overlapping in the 99th percentile of two (pairwise) cancer groups, Chi-squared analysis with Yates correction was performed.

### CulPRIT Selection

CulPRITs are genes of interest based on their repeated (pan-tumor) associations with the CD8+ T cell-depleted phenotype. Two selection strategies were employed to select CulPRITs. In the first, each gene's pan-tumor MPR (i.e., the median of a gene's percentile rank values across the 23 tumor groups) was computed based on the LFC and SC methods (in parallel). Genes with a MPR of ≥75 by both LFC and SC methods and also having a median FDR *q* value ≤0.1 by both methods were identified as MPR CulPRITs. In the second strategy, Bonferroni-corrected exact binomial probabilities were calculated to assess the significance of genes identified in the 99th percentile of ranked genes k out of 23 times (for 23 tumor groups). Genes that occurred in the 99th percentile of 5 or more of the 23 tumor groups were identified as EBP CulPRITs.

### Gene Enrichment Analyses

Gene ontology analyses were conducted using Ingenuity Pathway Analysis 2.4 (Qiagen) (26), the DAVID bioinformatics resource v6.7 (27), and the PANTHER statistical overrepresentation test v14.1 (28). Gene Set Enrichment Analysis (GSEA) (29) was performed using GSEA Desktop v3.0 and MSigDB v6.1 (http://software.broadinstitute.org/gsea/index.jsp).

### Mutual Exclusivity/Co-occurrence Analyses

Tumor RNAseq expression profiles were subset to comprise only those belonging to the T^SIG^-Low tertiles. CulPRIT genes were selected as indicated elsewhere. Gene expression values were mean-centered within tumor groups and binarized to low (below-mean = 0) or high (above-mean = 1) expression categories. Data were then concatenated across tumor groups for pan-tumor analysis. Fisher's exact test log_2_ odds ratios (ORs) and FDR-adjusted *q* values were computed for all pairwise gene combinations. Genes comprising pairwise combinations with significant ORs were selected using indicated cutoffs. Selected genes were clustered and visualized using Cluster 3.0 (30) (Spearman correlation similarity metric, complete linkage) and Java Treeview (31).

### Association of Gene Copy Number With Cytotoxic T-Lymphocyte Status

The somatic copy number alteration (SCNA) module of the Tumor IMmune Estimation Resource (TIMER) (32) was used to associate genetic copy number alterations of BMP7 with relative abundance of tumor-infiltrating CD8+ T cells.

### Expression Constructs and Cell Line Generation

Refer to the Supplementary Methods for detailed descriptions of cloning procedures and cell line generation.

### *In vivo* Mouse Studies

#### 4T1 Breast Cancer Model

2 × 10^5^ cells (4T1, 4T1-S, 4T1-S-pR26, or 4T1-S-pR26-mBMP7) were injected s.c. into the right fourth mammary fat pad of 6- to 8-week-old female BALB/c mice (Jackson Laboratories). Mice inoculated with 4T1-S-pR26 and 4T1-S-pR26-mBMP7 cell lines were administered 2 μg/ml doxycycline hyclate in drinking water containing 5% sucrose (w/v) and allowed to drink *ad libitum*. Control mice were given sucrose water. Tumors were measured longitudinally via caliper [volume formula: V = ((L+W)/2)^*^L^*^W^*^0.52)], and tumors were harvested at 2, 3, and 4 weeks (*n* = 5 mice per group, per time point) for anti-CD8a immunofluorescence analysis.

#### MC38 Colon Adenocarcinoma Model

1 × 10^6^ cells (MC38-pR26-CMVconst or MC38-pR26-CMVconst-mBMP7) were injected s.c. into the right flank of 6- to 8-week-old female C57BL/6 mice (Envigo). On days 8, 12, 16, and 20, post-inoculation mice were administered i.p. injections of either 10 mg/kg anti-PD-L1 (Bio X Cell cl. 10F.9G2) or isotype control antibody (Bio X Cell cl. LTF-2). Tumors were measured longitudinally via caliper, and tumors were harvested at 5 weeks for anti-CD8a immunofluorescence analysis and flow cytometry analysis of TILs (*n* = 10 mice per group). Note: refer to the Supplementary Methods for a detailed description of immunofluorescence and flow cytometry analyses.

#### Ethics Approval Statement

All animal experiments were approved by the Institutional Animal Care and Use Committee (IACUC) of Wake Forest University (protocol no. A16-045) and were conducted in accordance with the NIH guidelines for the care and use of laboratory animals.

### Single Cell RNAseq Analysis

4T1-S-pR26-CMVconst control and BMP7-expressing tumors were harvested at 3 weeks post-inoculation. Tumor fragments were collagenase-digested, and single cell suspensions were purified by filtering and density gradient centrifugation (as described in the Supplementary Methods). Single cell cDNA libraries were prepared using a 10X Genomics Chromium Controller, and indexed libraries were paired-end sequenced on an Illumina NextSeq 500 at a targeted read depth of 100,000 reads per cell. Raw bcl and fastq data were demultiplexed, normalized, and post-processed using R-based CellRanger mkfastq pipelines and QC algorithms. The machine learning technique t-distributed stochastic neighbor embedding (t-SNE) was used to reduce data dimensionality and cluster cells based on global gene expression patterns. The Loupe Cell Browser (10X Genomics) was used to examine cluster-specific gene expression and compute FDR-adjusted *p* values for differentially expressed genes.

## Results

### Genes Upregulated in CD8-Low Tumors Are Conserved Across Cancer Types

**Figure 1:**
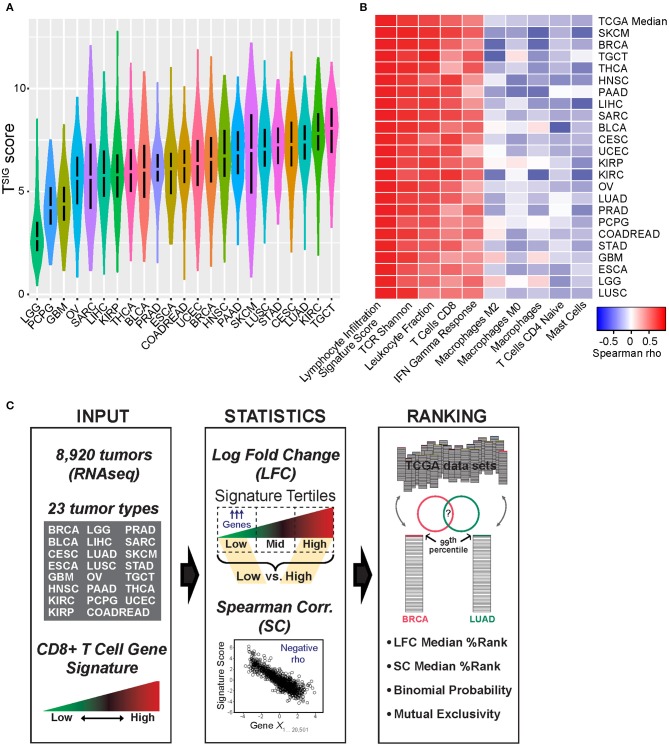
Characterization of T^SIG^ and schematic of approach. **(A)** The distribution of T^SIG^ metagene scores within The Cancer Genome Atlas (TCGA) tumor groups is shown. **(B)** Within tumor groups, T^SIG^ scores were compared by Spearman rank correlation (SC) to various tumor immunological measures as previously defined and annotated by Thorsson et al. (23), including CIBERSORT (34) immune cell proportion estimates. Shown are correlates selected from the most positive and negative pan-tumor associations. **(C)** Schematic of bioinformatics approach applied to candidate protein regulator of immune trafficking (CulPRIT) discovery and ranking. *Input:* T^SIG^ and C^SIG^ metagene scores are used to quantify relative T cell infiltration levels using tumor RNAseq profiles spanning 23 tumor types. *Statistics:* Metagene scores are used to perform log fold change (LFC) analysis of differentially expressed genes (low vs. high signature tertiles) and, in parallel, SC analysis to identify genes negatively correlated to metagenes. *Ranking:* Genes are assigned percentile ranks within tumor groups based on LFC and SC analyses. The top 1% (99th percentile) of ranked genes are compared across tumor types. CulPRITs are defined as the subset of genes that, across tumor types, show consistent or significant associations with a CD8-Low tumor phenotype. Using LFC and SC ranks, median percentiles and exact binomial probability are used in parallel to rank and independently define CulPRITs. Mutual exclusivity and pathway analyses are applied for the further characterization of CulPRITs.

We investigated the relationship between tumor gene expression patterns and a measure of tumor-infiltrating CD8+ T cells to identify genes (CulPRITs) recurrently associated with a CD8-Low tumor phenotype. CD8+ T cell abundance was quantified from tumor RNAseq profiles using a four-gene expression signature score (referred to as T^SIG^) derived from the geometric mean of the normalized log_2_ read counts for the genes CD8A, CD8B, CD3D, and CD3E. In parallel to T^SIG^, which reflects cell identity, we also considered a second gene signature reflective of cytotoxic T-lymphocyte (CTL) and/or NK cell cytolytic activity (referred to as C^SIG^), consisting of the genes GZMA, GZMB, GNLY, and PRF1. We computed T^SIG^ and C^SIG^ scores for 8,920 TCGA tumors grouped according to 23 solid tumor types. Notably, within tumor groups, we observed that the T^SIG^ and C^SIG^ scores were highly significantly and consistently correlated with each other, as well as correlated to reported gene signatures that reflect T cell abundance in tissues (Supplementary Figure 1; Supplementary Table 1). Furthermore, T^SIG^ and C^SIG^ scores were both broadly associated with favorable patient survival in the TCGA cohorts after adjusting for clinical variables (Supplementary Figure 2) and were significantly and consistently associated with histologic TIL abundance in TCGA samples as assessed by pathology review and histological scoring, defined in Saltz et al. (33) (Supplementary Figure 3). (Note that, given the high degree of correlation between T^SIG^ and C^SIG^, we report T^SIG^ results in the main figures and C^SIG^ results in the Supplementary Datasheet 2.) While T^SIG^ scores varied by magnitude across tumor types (Figure 1A), they exhibited consistent positive or negative correlations with certain other immunological measures previously annotated for TCGA tumors (23) (Figure 1B). Across cancer groups, T^SIG^ scores were consistently positively correlated with the lymphocyte infiltration signature, TCR Shannon index (a measure of T cell receptor clonal diversity), and signatures of CD8+ T cells and IFN gamma response, confirming the positive association between T^SIG^ and a T cell-inflamed tumor phenotype. Additionally, and to a lesser extent, T^SIG^ was negatively correlated with signatures of Mast cells, CD4+ naïve T cells, and M0 and M2 macrophages (Figure 1B).

**Figure 2:**
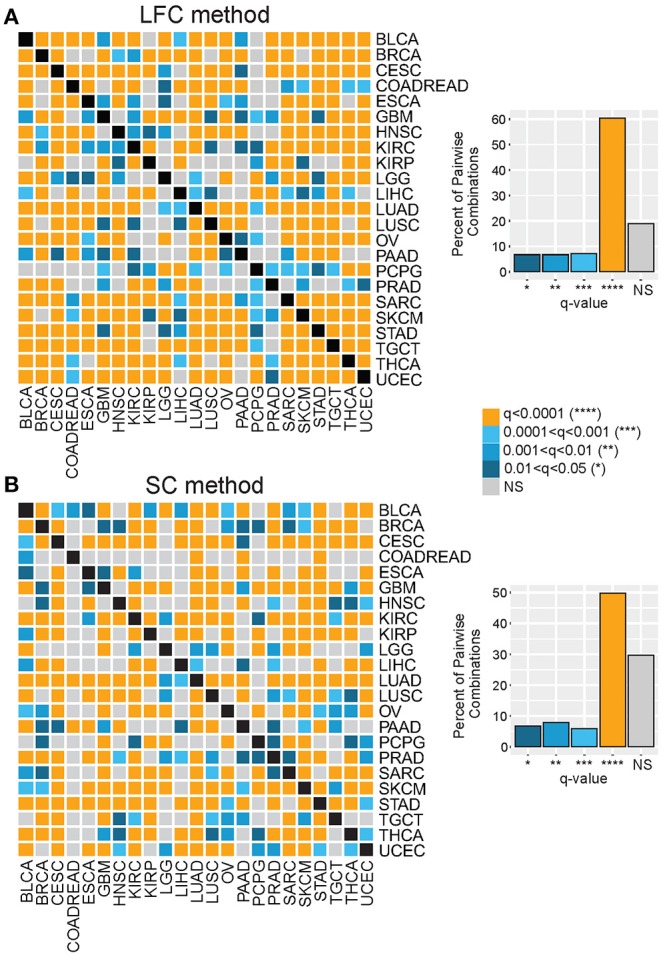
Genes associated with the T cell–cold phenotype are shared across diverse cancer types. Genes comprising the top (99th) percentile rank for each of 23 tumor types were compared by Chi-squared test for all pairwise tumor group combinations. Heat maps show the significance (see color key) of overlapping 99th percentile genes for each tumor group pairwise combination, where gene ranking was based on **(A)** the LFC method or **(B)** the SC method. Bar charts display the percent of pairwise comparisons that achieved statistical significance (*q*, FDR-corrected *p* values) at indicated thresholds for each method.

As depicted in Figure 1C, we investigated gene expression patterns inversely associated with T cell abundance by two parallel methods: (1) differential gene expression, referred to as the LFC method, and (2) Spearman correlation, referred to as the SC method. For the LFC method, within cancer types, tumors were stratified on signature score and then categorized into lower, intermediate, and upper signature tertiles—corresponding to a relative measure of CD8-Low, CD8-Intermediate, and CD8-High tumor subgroups. Gene expression levels were then compared between CD8-Low and CD8-High tumors to calculate the average LFC in gene expression for each of the 20,501 genes annotated in the RNAseq data matrix. Genes were then rank-ordered by their LFC, and this ordering was normalized across cancer groups as gene *percentile ranks*, with higher percentile ranking indicative of genes more highly overexpressed in CD8-Low tumors vs. CD8-High tumors. Using the SC method, within cancer types, the expression of each gene was tested for correlation to the signature score (irrespective of tertiles), and the resulting Spearman rho values were used to rank-order genes and compute their percentile ranks. By this method, higher percentile ranking is indicative of genes more negatively correlated with signature score. We then compared gene ranks across the different cancer types to test the hypothesis that transcriptional characteristics of CD8-Low tumors are shared among cancers of different anatomical origin. First, we analyzed all pairwise combinations of the 23 cancer types, comparing the top percentile of ranked genes identified in each cancer type (i.e., the 99th percentile of genes defined by either LFC or SC, *n* = 205 genes), and determined the significance of overlapping genes by Chi-squared test (Figure 2, Supplementary Figure 4). Strikingly, we observed that the large majority of pairwise comparisons between one tumor type and another showed statistically significant overlap among the 99th percentile genes after false discovery correction (*q* < 0.05). This observation held true for genes ranked by either LFC or SC methods, or according to T^SIG^ or C^SIG^ [81 and 70.4% of pairs overlap at *q* < 0.05 for T^SIG^ LFC and SC methods, respectively (Figure 2); 90.9 and 85.8% of pairs overlap at *q* < 0.05 for C^SIG^ LFC and SC methods, respectively (Supplementary Figure 4)]. These findings suggest that the transcriptional programming of T cell-cold tumors is, in part, composed of genetic features conserved across cancers in tumor-agnostic fashion and support the hypothesis that tumors exploit common transcriptional programs to regulate the intratumoral volume of CD8+ T cells.

### Biological and Transcriptional Characterization of CulPRITs

**Figure 3:**
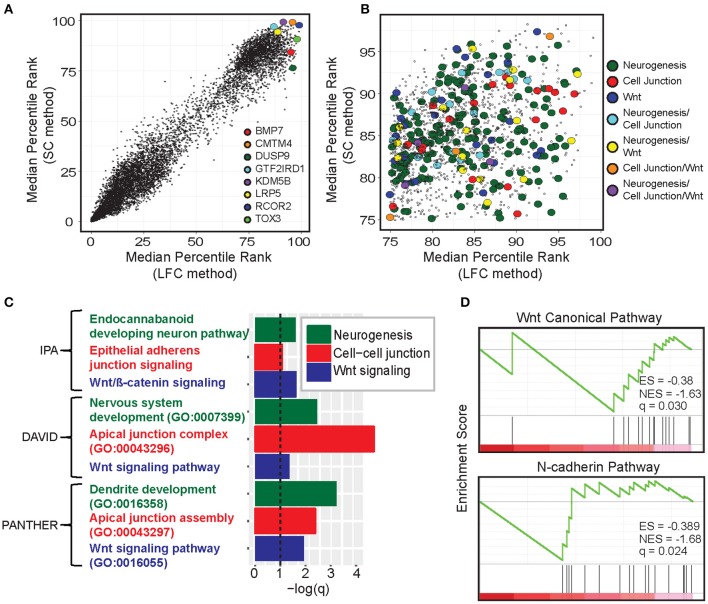
Gene- and pathway-level analysis of median percentile rank (MPR) CulPRITs. **(A)** Scatter plot of the pan-tumor MPRs of genes ranked by the LFC and SC methods. Genes with a median *q* > 0.1 (by either method) were omitted. Genes with interesting immunomodulatory functionality are highlighted (discussed in Supplementary Table 2). **(B)** Scatter plot of CulPRIT genes from **(A)** with MPRs ≥ 75 and annotated for involvement in enriched gene ontology categories. **(C)** Significant gene ontology categories identified among the CulPRIT genes by IPA, DAVID, and PANTHER algorithms are shown. **(D)** Gene Set Enrichment Analysis (GSEA) of CulPRIT genes shown for pathways related to Wnt signaling and cell-cell junction biology.

Next, we investigated the underlying biology of genes and pathways enriched in immunologically cold tumors. Here, we defined CulPRITs based on their cross-tumor MPRs (Figure 3). As each gene has a percentile rank in each tumor type, the MPR is the median of a gene's percentile ranks across the 23 tumor types. In this analysis, MPR CulPRITs were defined as the genes most consistently overexpressed in CD8-Low tumors by LFC (having LFC MPR ≥ 75th percentile) and, simultaneously, most inversely correlated with T^SIG^ score by SC (having SC MPR ≥ 75th percentile) (Figure 3B). Notably, a number of top CulPRITs having high SC and LFC MPRs have been implicated in pathways of immune modulation (Figure 3A, Supplementary Table 2) and include CMTM4 (promotes PD-L1 protein stabilization), BMP7 and LRP5 (promote alternative/anti-inflammatory macrophage polarization), TOX3 (transcription factor related to TOX, which regulates T cell development), and REST corepressor 2 (RCOR2), KDM5B, DUSP9, and GTF2IRD1 (involved in regulation of interferon and inflammatory signaling), indicating that genes reported to modulate immune signaling rank highly within our candidate gene pool. Gene ontology analysis of the MPR CulPRITs revealed enrichment of genes involved in biological processes related to *Wnt signaling, neurogenesis*, and *cell-cell junctions*, which were reproducibly identified using different ontology assessment algorithms (IPA, DAVID, and PANTHER, *q* < 0.1; Figures 3B,C). GSEA further verified the association of these genes with Wnt signaling and cell junction biology (N-cadherin pathway) (Figure 3D).

**Figure 4:**
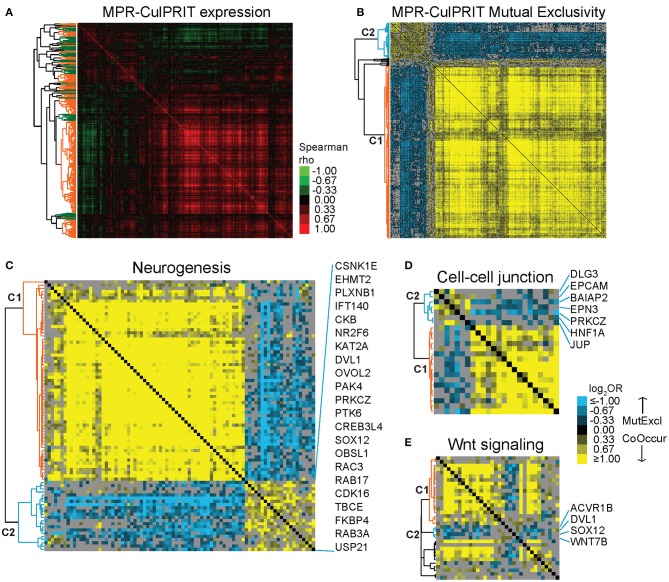
Mutual exclusivity analysis of MPR CulPRITs. We conducted pan-tumor correlation studies of CulPRIT genes (*n* = 1,417) identified by median MPRs. **(A)** Pan-tumor correlation matrix of MPR CulPRIT gene expression. Heat map colors reflect Spearman rho values. Colored dendrogram clusters (with average correlation of *R* ≥ 0.15) highlight synexpression groups (orange and green distinguish adjacent clusters). **(B–E)** Pan-tumor analysis of gene mutual exclusivity or co-occurrence was performed on CulPRIT gene subsets. Shown are clustered heat maps of gene-pair log_2_ odds ratios (ORs) derived from Fisher's exact test analysis. Blue denotes negative associations (mutual exclusion); yellow depicts positive associations (co-occurrence; see color key). Gray indicates sub-significant associations (*q* > 0.001). Genes comprising the major dendrogram clusters are indicated by cluster 1 (C1, orange branch) and cluster 2 (C2, blue branch). **(B)** Shown are the genes from **(A)** with highly significant involvement in any one pairwise gene combination having the threshold of log_2_OR < −1.0 (for mutual exclusion) and *q* < 1 × 10^−30^. Subsequent panels show similar heat maps for the subsets of **(C)** neurogenesis-annotated genes **(D)** cell-cell junction-annotated genes, and **(E)** Wnt signaling-annotated genes; shown are genes belonging to any one pairwise gene combination having the threshold of log_2_OR < −0.5 and *q* < 0.001. See Supplementary Table 4 and Supplementary Figure 5 for additional details.

Next, we studied the transcriptional properties of the MPR CulPRITs in CD8-Low tumors. Examination of the pan-cancer gene expression correlation structure demonstrated the existence of numerous synexpression groups, equating with 39 gene clusters with average correlation >0.15 (Figure 4A). However, the majority of clusters fell within one of two larger, inversely correlated gene clusters, whose inverse correlation suggested the possibility of transcriptional *mutual exclusivity* among CulPRITs in CD8-Low tumors. In cancer, multiple genetic alterations that confer the same selective advantage are not required; as such, different driver mutations that alter the same oncogenic pathway seldom occur within the same tumor, but display mutually exclusive occurrence patterns. Thus, mutually exclusive relationships tend to underlie functionally relevant, phenotypically conserved genetic alterations in cancer. To determine if CulPRIT expression profiles exhibit mutual exclusivity in CD8-Low tumors, we examined all pairwise combinations of CulPRITs for statistically significant mutual exclusion patterns. Shown in Figure 4B are the pairwise log_2_ ORs (Fisher's exact test) of MPR CulPRITs exhibiting the most significant relationships (log_2_OR < −1, *q* < 1 × 10^−30^; *n* = 341 genes). Two predominant gene clusters exhibiting highly significant mutual exclusivity emerged (clusters C1 and C2). Notably, the mutually exclusive relationship between the two clusters appeared largely independent of cancer type (Supplementary Table 4), and neither cluster displayed significant enrichment for specific biological processes or pathways according to gene ontology analysis. In fact, the genes comprising the previously identified enriched terms related to Wnt signaling, neurogenesis, and cell-cell junction were proportionally distributed among the two gene clusters (Figures 4C–E, Supplementary Figure 5), suggesting that these biological pathways likely do not independently explain these mutually exclusive transcriptional patterns. Analysis of individual gene pairs identified NR2F6 (a component of C2, Figures 4B,C) and ATF2 (a component of C1) as the most statistically significant mutually exclusive gene pair from a total of 1,003,236 CulPRIT pair combinations analyzed (OR = 0.23, *q* = 2.4 × 10^−77^, Fisher's exact test). Strikingly, both genes have been mechanistically associated with suppressive functions in tumor immune surveillance. While expression of NR2F6 (an orphan nuclear receptor) has been linked to malignant growth and progression in multiple cancer types, in tumor-reactive T cells, NR2F6 functions to repress expression of effector cytokines and acts as an intracellular immune checkpoint that inhibits CD8+ T cell infiltration and suppresses anti-tumor immune responses (35). ATF2 is a transcription factor with emerging regulatory roles in inflammatory signaling and was recently shown to inhibit IFNβ expression and type I interferon signaling in melanoma (36). Together, these findings suggest that transcriptional programs shared across tumors may reflect immunomodulatory mechanisms that contribute to the CD8+ T cell-depleted state.

**Figure 5:**
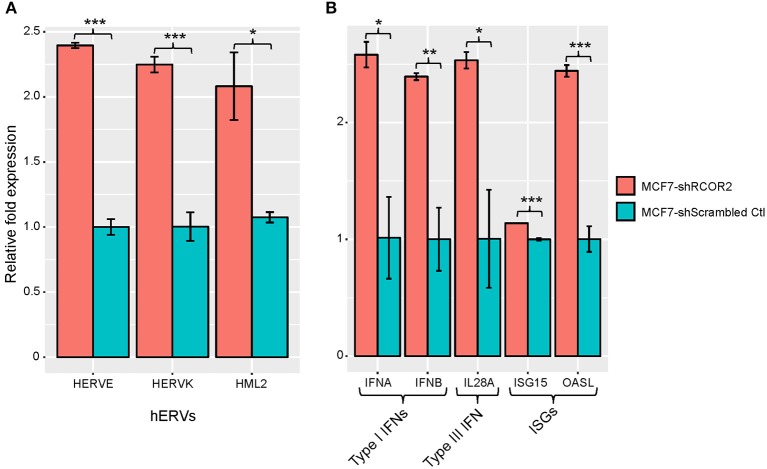
REST corepressor 2 (RCOR2) negatively regulates human endogenous retrovirus (hERV), IFN, and interferon-stimulated gene (ISG) expression. shRNA-mediated downregulation of RCOR2 in MCF7 cells resulted in significantly increased expression of **(A)** hERVs and **(B)** type I IFNs, type III IFN, and ISGs. Significance codes: *p < 0.05; **p < 0.01; ***p < 0.001 (Student's *t*-test). Error bars show the standard error of the mean (*n* = 3). Refer to Supplementary Table 5 for qPCR primer pairs used in this analysis.

In parallel to the above studies, we also considered a more statistically rigorous definition for CulPRITs. We computed the EBP that a gene, by random chance, would belong to the 99th percentile (by LFC or SC) of *k* out of 23 tumor types. Accordingly, we determined that the probability of identifying a single gene belonging to the 99th percentile in 5 out of 23 tumor types is *p* = 0.058. As shown in Table 1, one or more genes were identified at *k* values ranging from 5 to 12 tumor types, for a total of 90 and 63 genes identified in the top percentile rank of at least 5 of 23 tumor types by the LFC and SC methods, respectively (see also Supplementary Datasheet 1, Supplementary Table 3, and Supplementary Figure 6). We termed these genes EBP CulPRITs. Notably, the majority of these genes showed overlap with the MPR CulPRITs identified in the MPR studies (Figures 3, 4). The top CulPRIT identified in this analysis, as well as being a top CulPRIT identified in Figure 3A, was RCOR2. RCOR2 encodes an epigenetic modifier that binds to and promotes the H3K4 demethylation activity of LSD1/KDM1A (37). In a recent report, LSD1 ablation in a melanoma tumor model increased CTL infiltration into tumors and reversed ICB resistance (38). Mechanistic studies using MCF7 cells showed that LSD1 ablation de-repressed human endogenous retrovirus (hERV) expression, which induced dsRNA stress and subsequent expression and activation of type I and type III IFNs and interferon-stimulated genes (ISGs) (38). We therefore sought to determine if RCOR2 silencing in MCF7 cells would phenocopy LSD1 ablation. As shown in Figure 5, shRNA-mediated knockdown of RCOR2 resulted in significant transcriptional upregulation of hERVs, type I and III IFNs, and ISGs, consistent with the hypothesis that RCOR2 upregulation in tumors promotes a T cell-depleted phenotype by facilitating LSD1-mediated suppression of IFN signaling.

**Table 1:** Exact binomial probabilities associated with the discovery and ranking of candidate protein regulators of immune trafficking (CulPRITs).

k^*^ out of 23	Bonferroni p^**^	**LFC (no. genes)**	**Gene**	**SC (no. genes)**	**Gene**
12	2.48 × 10^−14^	1	*RCOR2*	0	–
11	2.46 × 10^−12^	0	–	0	–
10	2.06 × 10^−10^	1	*FREM2*	0	–
9	1.46 × 10^−8^	2	*CASKIN1, TOX3*	1	*CMTM4*
8	8.65 × 10^−7^	1	*SLC6A10P*	5	*ACVR2B, AKAP1, MAP7, RCOR2, TOM1L1*
7	4.28 × 10^−5^	8	*BMP7, DUSP9, EEF1A2, KIF1A, PPARGC1A, RAP1GAP, SLC15A1, SOX11*	14	*ACACA, ARHGAP32, CLDN12, FAM168B, GPR125, GTF2IRD1, HOOK1, KIAA1804, MAGI1, NCKAP1, PPME1, SUN1, ZCCHC14, ZFYVE9*
6	1.74 × 10^−3^	24	*ATP2C2, B4GALNT4, CA12, CECR2, CHP2, COL2A1, COL9A3, CREG2DLX2, FSTL4, LPPR1, MAGEA6, MAGEA9B, MYT1, PKP1, PLA2G4F, RPS6KA6, SLITRK6, SOSTDC1, SPRR1B, WDR72, WNT7B*	14	*CAMSAP1L1, EPCAM, EXTL2, F11R, FASN, KCTD3, KIAA1549, LRP6, POMT2, RAP1GAP, XPO5, ZNF74, CASKIN1, GTF3C2*
5	5.76 × 10^−2^	53	*AGR2, AR, C1QL4, CA9, CACNA1D, CACNG4, CAPN9, COL25A1, CPLX2, DLX3, DNAH2, DSG1, EREG, ESM1, FAM155B, FBN3, FIBCD1, GJB6, GPX2, GREB1, GRHL3, HMGA2, IYD, KIAA1244, KRT17, LGALS7B, LGR5, MAGEA2, MAGEA3, MUM1L1, NOTUM, ODZ2, OPRK1, PHGR1, PNCK, PTPRT, RAB3B, RASEF, ROBO2, SHANK2, SNORD116-4, SOX3, SPRR1A, SSPO, ST8SIA2, SYT13, SYT7, SYTL5, TFF3, TMEM38A, UNC5D, UPK1B, ZIC2*	29	*ACLY, C19orf26, C1orf27, CYP51A1, DDR1, DHCR7, EPB41L5, ESRP1, FNBP1L, HMGCR, HMGCS1, ICA1, KDM4B, KIAA1543, LCLAT1, LRP5, MIPOL1, MYEF2, PLA2G12A, RMND5A, TTC3, ZKSCAN2, ADNP, C14orf128, KDM5B, L2HGDH, MYO10, PCYOX1, TET3*

* *The number of times that a gene falls into the top percentile rank out of 23 tumor types*.

** *The likelihood of any one gene falling into the top percentile rank k out of 23 times*.

*LFC, log fold change; SC, Spearman correlation*.

**Figure 6:**
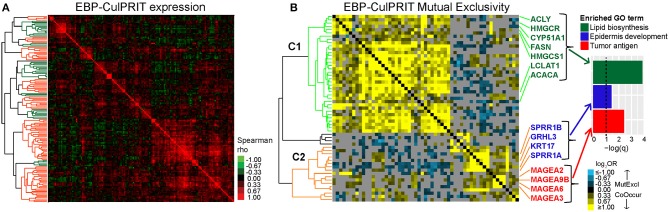
Mutual exclusivity analysis of exact binomial probability (EBP) CulPRITs. We analyzed the pan-tumor correlation structure of the 150 EBP CulPRIT genes (defined by EBP analysis) in CD8-Low tumors. **(A)** Pan-tumor correlation matrix of EBP CulPRIT gene expression. Heat map colors reflect Spearman rho values. Colored dendrogram clusters (with average correlation of *R* ≥ 0.15) highlight synexpression groups (orange and green distinguish adjacent clusters). **(B)** Pan-tumor analysis of gene mutual exclusivity or co-occurrence was performed. Shown is a clustered heat map of gene-pair log_2_ odds ratios comprised of the subset of genes from **(A)** that showed significant involvement in any one pairwise gene combination having the threshold of log_2_OR > −1.5 and *q* > 0.001. Blue denotes negative associations (mutual exclusion); yellow depicts positive associations (co-occurrence; see color key). Gray indicates sub-significant associations (*q* > 0.05). Genes comprising the predominant dendrogram clusters are indicated by cluster 1 (orange branch) and cluster 2 (green branch). Gene ontology analysis of genes comprising cluster 1 or 2 revealed the significant enrichment of lipid biosynthesis (cluster 1) and epidermal development and tumor antigenicity (cluster 2). GO term-associated genes are highlighted.

To gain further insight into the expression dynamics of the EBP CulPRITs, we analyzed their pan-tumor expression correlation structure. Similar to the analysis of Figure 4A, we observed numerous gene synexpression groups equating with 13 gene clusters with average correlation >0.15 (Figure 6A). Mutual exclusivity analysis resulted in 57 genes with highly significant mutually exclusive or co-occurrence relationships (Figure 6B) that mirrored the two predominant mutually exclusive CulPRIT clusters identified in Figure 4B. However, in this analysis, the mutually exclusive gene clusters coincided with different significantly enriched gene ontologies. Cluster 1 genes showed significant enrichment for the gene ontology term *lipid biosynthesis* (FASN, ACACA, ACLY, HMGCR, HMGCS1, CYP51A1, and LCLAT1), while cluster 2 genes showed enrichment for the terms *epidermal development* (GRHL3, KRT17, SPRR1A, and SPRR1B) and *tumor antigen* (MAGEA2, MAGEA3, MAGEA6, and MAGEA9B) (Figure 6B), suggesting possible roles for these biological processes in the promotion of the T cell-cold phenotype.

### A CulPRIT Gene Signature Is Associated With Immunotherapy Response and Survival

**Figure 7:**
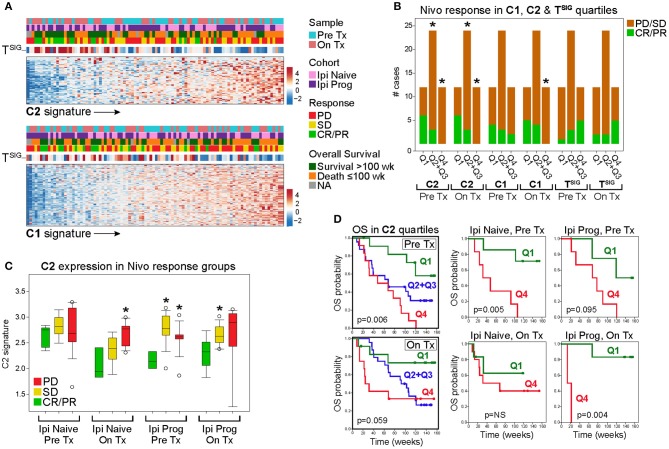
The C2 signature of CD8-Low tumors is associated significantly with nivolumab (Nivo) response and survival of melanoma patients. Genes comprising the MPR CulPRIT C1 and C2 clusters (*n* = 278 and *n* = 57 genes, respectively) described in Figure 4B were analyzed for associations with melanoma response and patient survival following Nivo treatment (Tx). The RNAseq data comprise 96 samples corresponding to Pre Tx and On Tx biopsies from patients who progressed on ipilimumab (Ipi Prog) or received no prior ipilimumab Tx (Ipi Naïve). **(A)** Heat map expression profiles of C1 and C2 signature genes (rows) in tumor samples (columns, oriented left to right by ascending signature score) are shown in association with the CD8+ T^SIG^ signature and patient clinical correlates (colored categories). **(B)** Profiles of Pre Tx (*n* = 48) and On Tx (*n* = 48) biopsies were sorted into C1, C2, and T^SIG^ signature quartiles (Q) to examine associations with patient response. CR, complete response; PR, partial response; PD, progressive disease; SD, stable disease. *p < 0.05, Fisher's exact test, indicated Q group compared to Q1. **(C)** C2 signature score distributions within patient response groups are shown according to Tx cohort and biopsy type. *p < 0.05, Fisher's exact test, indicated response group compared to CR/PR. **(D)** C2 quartile groups were compared for overall survival (OS) in Pre and On Tx samples (left Kaplan-Meier plots) and within Tx cohorts and biopsy types (right Kaplan-Meier plots). Log-rank *p* values are shown.

The mutually exclusive gene clusters identified in CD8-Low tumors may reflect biological signatures of immune escape that correlate with poor response to immunotherapy. To test this, we leveraged the RNAseq data set of Riaz et al. (24), who profiled melanoma biopsies of nivolumab (Nivo)-treated patients who either progressed on ipilimumab (Ipi Prog) or were ipilimumab-naïve (Ipi Naïve) prior to Nivo treatment. Gene signature scores based on the C1 and C2 clusters from MPR CulPRITs (see Figure 4B, the largest of the mutually exclusive clusters identified, comprising 278 and 57 genes, respectively) were computed for both pre-treatment and on-treatment tumor biopsies and analyzed for associations with treatment response and overall survival (Figure 7A). As anticipated, both C1 and C2 signatures were found to be inversely correlated with the T^SIG^ signature in this data set (C1: rho = −0.33, *p* = 9.3 × 10^−3^; C2: rho = −0.49, *p* = 6.4 × 10^−7^). Pre-treatment and on-treatment tumor samples were classified into C1 and C2 signature quartiles (Q) and analyzed for associations with Nivo response (Figure 7B). In both pre- and on-treatment biopsies, the C2 signature, in particular, showed significant associations with Nivo response. In both pre- and on-treatment data sets, two-thirds of responding patients (CR/PR) were classified into the C2 signature low expression quartile (Q1), while one-third and zero responding patients were classified into the C2 interquartile range (Q2+Q3) and the C2 high expression quartile (Q4), respectively (Q1 vs. Q4, *p* = 0.014, and Q1 vs. Q2+Q3, *p* = 0.036, Fisher's exact test). Associations with the C1 signature quartiles were notably weaker, with significance achieved only in the on-treatment biopsies for Q1 vs. Q4 (*p* = 0.037, Fisher's exact test). Of note, T^SIG^ quartiles exhibited a positive association with response, as compared to the negative association observed of C1 and C2. However, the T^SIG^ association did not reach significance (Figure 7B). The C2 signature also showed consistent expression differences between patient response groups (CR/PR, SD, PD) in both Ipi Naïve and Ipi Prog cohorts (Figure 7C), as well as significant and near-significant associations with patient overall survival in Ipi Naïve (pre-treatment) and Ipi Prog (pre- and on-treatment) cohorts, despite small patient numbers (Figure 7D). Together, these findings demonstrate significant relationships between C2 gene expression level in melanoma biopsies and clinical outcomes following anti-PD1 treatment.

### BMP7 Limits Abundance of Tumor-Infiltrating CD8+ T Cells

**Figure 8:**
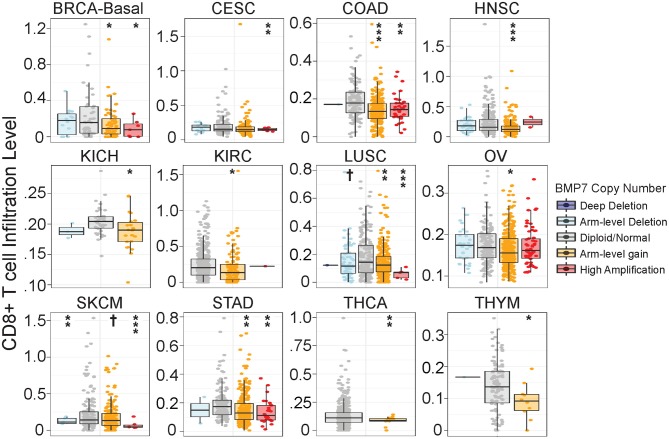
Bone morphogenetic protein 7 (BMP7) copy number gain is associated with significant reduction in CD8+ T cell infiltration across cancer types. Analysis of somatic copy number alterations associated with gene signature estimates of CD8+ T cell infiltration as described in Li et al. (32). BMP7 copy number was quantified by GISTIC 2.0 (40). The infiltration level for each copy number category is compared with normal ploidy using two-sided Wilcoxon rank sum test. Significance codes: †*p* < 0.1; *p < 0.05; **p < 0.01; ***p < 0.001.

**Figure 9:**
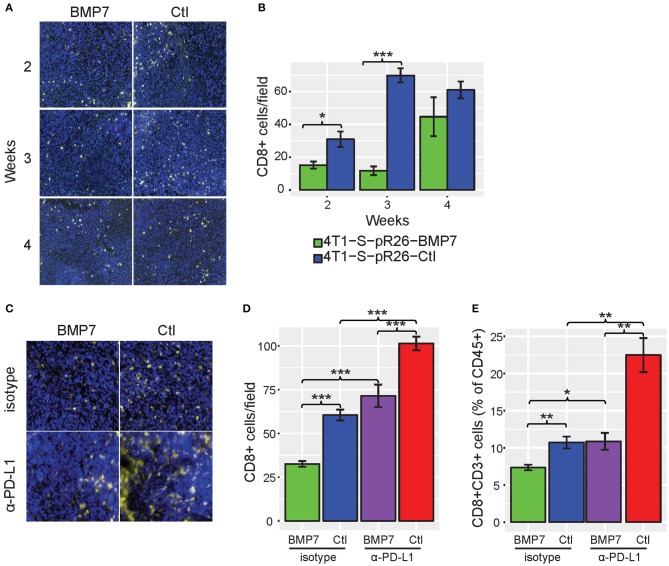
BMP7 limits T cell abundance in mouse tumor models. **(A)** Representative immunofluorescent images of CD8+ T cell staining [CD8 (yellow) and DAPI (blue)] in control vs. BMP7-expressing mouse 4T1-S tumors harvested at 2, 3, and 4 weeks post-tumor cell implantation. **(B)** CD8+ T cell abundance in 4T1-S tumors by CD8 staining (*n* = 5 tumors per condition, per time point; five to six random fields per section were counted). **(C)** Representative immunofluorescent images of CD8+ T cell staining [CD8 (yellow) and DAPI (blue)] in control vs. BMP7-expressing mouse MC38 tumors treated with anti–PD-L1 or isotype control antibody, harvested at 5 weeks post–tumor cell inoculation. **(D)** CD8+ T cell abundance in MC38 tumors by CD8 staining (*n* = 10 animals per group; five to six random fields per section were counted). **(E)** CD8+ T cell abundance in MC38 tumors by flow cytometry assessment and as a percentage of CD45+ cells. Significance codes: *p < 0.05; **p < 0.01; ***p < 0.001 (Student's *t*-test). Error bars show the standard error of the mean.

As a number of our top CulPRIT genes have known roles in immune modulation, we reasoned that some may have previously unidentified functions in T cell exclusion. To examine this possibility, we performed phenotypic assays on one of our top CulPRITs, BMP7 (Figure 3A, Table 1). BMP7 is a TGF-β homolog whose family members are known to modulate immune responses, including the promotion of immunosuppression (39). BMP7 has reported immunomodulatory roles that include regulation of monocyte adhesion and migration, antagonism of inflammatory cytokine production, and promotion of alternative M2 macrophage polarization (Supplementary Table 2). In our study, BMP7 ranked in the top percentile of LFC-ranked tumors in 7 out of 23 cancer types (*p* = 4.28 × 10^−5^; Table 1). Moreover, we observed that amplification of the BMP7 gene locus is significantly associated with reduced CD8+ T cell infiltrates across a range of cancers (Figure 8) and is upregulated in immune-privileged organs such as the brain and placenta (Supplementary Figure 7). BMP7 has been reported to be overexpressed by the malignant epithelial cells of some breast and colorectal tumors (41, 42). Therefore, to test if overexpression of BMP7 can promote a T cell-cold state, we exogenously expressed BMP7 in murine 4T1-S (breast) and MC38 (colon) cancer cells (both of which lack endogenous BMP7 expression) and studied the *in vivo* effects of BMP7 on intratumoral CD8+ T cell abundance. The murine 4T1-S cell line is an immunogenic variant of the syngeneic triple negative breast cancer cell line, 4T1 (43) (Supplementary Figures 8–10). BMP7-expressing and control tumors were grown in the mammary fat pad of BALB/c mice and examined for CD8+ T cell infiltration during tumor growth. Immunofluorescence analysis of tumor sections showed a significant BMP7-dependent reduction in CD8+ T cells at both the 2- and 3-week time points (Figures 9A,B; *p* = 0.012 and *p* < 0.001, respectively). Using the MC38 immunogenic colon cancer model, BMP7-expressing and control tumors were grown in the flanks of C57BL/6 mice treated with anti-PD-L1 or isotype control antibody, and examined for CD8+ T cell infiltration at 5 weeks post-inoculation (Supplementary Figures 8, 9, 12). By immunofluorescence and flow cytometry analysis, a significant BMP7-dependent decrease in CD8+ T cell abundance was observed in tumors of mice treated with the isotype control antibody (*p* < 0.01, Figures 9C–E). In tumor-bearing mice treated with anti-PD-L1, a significant increase in tumor-infiltrating CD8+ T cells was observed in both experimental groups (*p* < 0.05). However, the anti-PD-L1-mediated increase in CD8+ T cell numbers was significantly attenuated by BMP7 expression (*p* < 0.01; Figures 9D,E).

**Figure 10:**
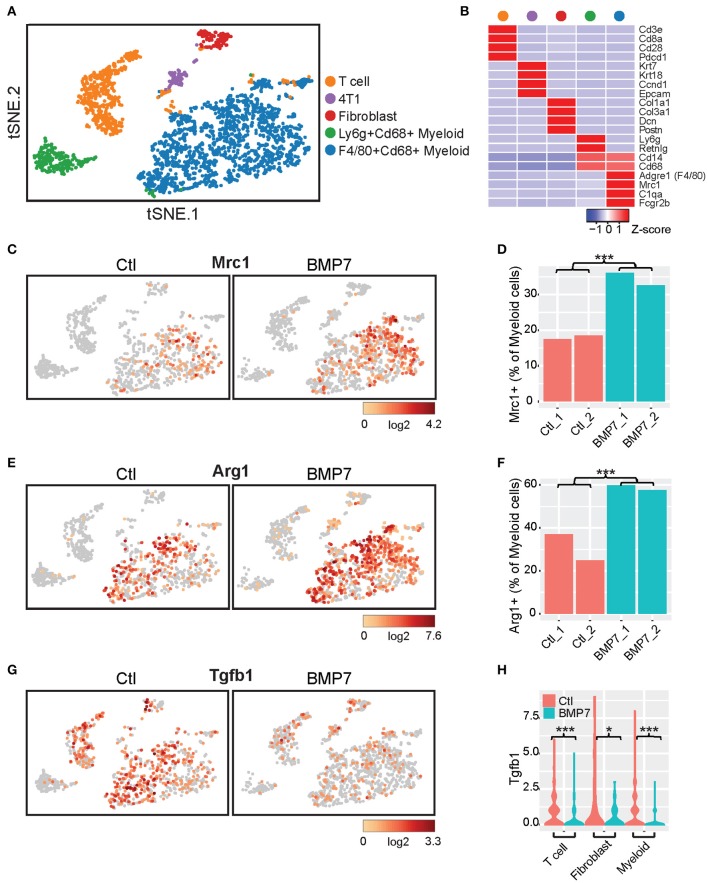
Single cell RNAseq analysis of BMP7-expressing and control 4T1-S tumors. **(A)** t-distributed stochastic neighbor embedding (t-SNE) plot of K-means-clustered tumor cell populations representing two control (Ctl) and two BMP7-expressing (BMP7) tumors (*n* = 2,519 cells, total). **(B)** Z-score-normalized expression of gene markers that uniquely define the cell clusters depicted in **(A)**. **(C,E,G)** Shown are t-SNE plots illustrating the relative cell expression levels of **(C)** Mrc1, **(E)** Arg1, and **(G)** Tgfb1. **(D,F)** Bar plots of the percentage of tumor-infiltrating myeloid cells positive for expression of **(D)** Mrc1 and **(F)** Arg1. ***p < 0.001 (Chi-squared test). **(H)** Violin plots of Tgfb1 expression distributions in tumor-infiltrating T cells, fibroblasts, and myeloid cells. *p < 0.05; ***p < 0.001 (Student's *t*-test).

Interestingly, no difference in tumor growth was observed between the BMP7-expressing and control tumor groups of the 4T1-S model (Supplementary Figure 11), and BMP7 expression in the MC38 model did not decrease the efficacy of anti-PD-L1, but rather, in a somewhat paradoxical fashion, the combination of BMP7 expression and anti-PD-L1 resulted in a significant reduction of tumor growth (Supplementary Figures 12B,C). In cancer cells, BMP7 has both oncogenic and tumor suppressor-like functions (44) and is known to antagonize the TGF-β signaling axis (45). Single cell RNAseq analysis of 4T1-S BMP7-expressing and control tumors revealed that while BMP7 expression is associated with increased Mrc1 (Cd206) and Arg1 myeloid-specific expression (*p* < 0.001; Figures 10A–F) consistent with BMP7's role in M2 macrophage polarization, the single most differentially expressed gene was Tgfb1. In BMP7-expressing tumors, Tgfb1 was significantly and simultaneously decreased in myeloid cells (*p* < 0.001), cancer associated fibroblasts (*p* < 0.05), and T cells (*p* < 0.001) (Figures 10G,H). This suggests that while BMP7 may act to limit T cell abundance in tumors, a TME-wide repression of Tgfb1 (mediated by BMP7) could alleviate Tgfb1-dependent immunosuppression and in turn promote anti-PD-L1 efficacy. Together, these results support the hypothesis that BMP7 expression in tumors may negatively regulate CD8+ T cell infiltration, but with variable phenotypic effects on tumor growth. These findings support the credibility of our CulPRIT discovery platform for identifying novel drivers of the T cell-cold state.

## Discussion

In this study, we describe the development and implementation of a large-scale pan-cancer informatics platform to shed light on the transcriptional programming that underlies the immunologically cold tumor state. A novel and key finding in this study was the highly significant and pervasive overlap of CD8-Low associated genes (CulPRITs) found among tumors of diverse tissue origin. Indeed, just as certain driver mutations are repeatedly selected for in tumors of diverse tissue origin, this finding may indicate that the pathologic upregulation of certain genes may be broadly acquired by tumors to facilitate immune evasion by T cell exclusion. Among the CulPRITs identified were genes enriched for biological pathways with immunomodulatory implications. Wnt/β-catenin signaling, identified in our analysis, is a confirmed driver of T cell exclusion in melanoma (46). Wnt/β-catenin signaling in melanoma cells promotes immunological tolerance by limiting DC maturation, promoting IDO production, and suppressing IFNγ production by CTLs (47, 48). Our finding that genes involved in Wnt/β-catenin signaling are enriched in the CD8-Low tumor phenotype in multiple tumor types corroborates a recently published study implicating this pathway in immune exclusion in different solid tumors (49), thereby supporting the potential of our approach to discover functionally relevant gene pathways.

Genes involved in neurogenesis were also enriched among CulPRITs. This finding is intriguing, as the central nervous system (CNS) has traditionally been viewed as an immune-privileged site (50). While a more modern understanding of the CNS suggests that its immune privilege may be context dependent, it is widely believed that at baseline homeostasis, the CNS promotes a subdued state of immune surveillance in order to reduce the potential inflammatory reactions that could otherwise cause neuronal bystander damage (51). Our finding that CD8-Low tumors are enriched for genes associated with neurogenesis may reflect the repurposing of CNS-specific gene transcription by tumors to induce a CNS-like anti-inflammatory state that facilitates immune evasion. Further studies to confirm the activation of CNS-specific transcriptional networks in tumors and their subsequent impact on the immune microenvironment are warranted.

Our findings also point to a role for cell-cell junctions in tumor T cell exclusion. The importance of cell junction integrity and regulation of inflammation is well-documented in models of experimental colitis. Immuno-pathological conditions such as inflammatory bowel disease are associated with a dysfunctional epithelial barrier that results in increased leukocyte recruitment and inflammation (52). Tight junction dysfunction that gives rise to leaky intestinal walls can promote colorectal cancer development through induction of a chronically inflamed state (52). In this study, observation of an inverse association between cell junction biology and tumor inflammation suggests that our findings may relate to transcriptional programs that strengthen cell-cell junctions or impede transcellular migration of T cells resulting in the suppression of T cell penetration into tumors.

Intriguingly, we observed evidence of mutually exclusive transcriptional programs among the MPR CulPRITs. This suggests the existence of parallel mechanisms that may be exploited by tumors to actuate the immune-cold phenotype. At the individual gene level, the nuclear orphan receptor NR2F6 together with the transcription factor ATF2 emerged as the most significant mutually exclusive gene pair in pan-tumor analysis. Consistent with this observation, NR2F6 has been documented to function as a gatekeeper of anti-tumor immunity through transcriptional repression of proinflammatory cytokines including IL-2, IFNγ, and TNFα (35, 53). Furthermore, NR2F6 inhibition has been shown to augment the efficacy of ICB in preclinical prostate, melanoma, and colorectal cancer models, as well as increase tumor infiltration by IFNγ-positive T cells (35, 54). However, an immunoevasive function for NR2F6 cancer-specific expression has not yet been described. ATF2 is a leucine zipper transcription factor with documented roles in inflammatory pathologies such as obesity, inflammation-induced pain, hepatitis, and asthma, and its known functions include transcriptional regulation of CAMs, proinflammatory cytokines, and chemokines (55). Importantly, an anti-inflammatory role of ATF2 in cancer has recently been established whereby PKCε-mediated activation of ATF2 directly inhibits IFNβ expression and type I interferon signaling in melanoma (36). As type I interferon signaling in cancer is a potent inducer of anti-tumor immune surveillance, our findings are consistent with a role for ATF2-mediated suppression of IFNβ in the T cell exclusion phenotype. Other notable genes that displayed mutually exclusive expression patterns in T cell-cold tumors include the Wnt signaling gene DVL1 (Figure 4C) and the cell-cell junction-annotated genes BAIAP2 and PRKCZ (Figure 4D). DVL1 has been reported to decrease CD8+ T cell abundance in intestinal tissues and suppress activation and degranulation of trafficking T cells (56). BAIAP2 and PRKCZ have inflammation-suppressing functions. BAIAP2 has been shown to control the expression of the proinflammatory cytokines IL-6 and IL-1α (57), and PRKCZ has been noted to play a role in controlling inflammatory dermal mesenchymal stem cells in psoriasis, a T cell-mediated disease (58).

Intriguingly, the mutually exclusive gene clusters observed among the EBP CulPRITs showed differential enrichment for genes associated with the GO terms *lipid biosynthesis* (Figure 6B, cluster 1), *epidermal development*, and *tumor antigens* (Figure 6B, cluster 2). The FASN gene (*lipid biosynthesis*) of cluster 1 encodes a key metabolic enzyme in lipogenesis whose elevated expression has been associated with intratumoral T cell depletion in advanced-stage ovarian cancer (59). This observation was recapitulated in a murine ovarian cancer model, where FASN overexpression reduced TIL numbers and impaired antigen presentation by dendritic cells, resulting in defective T cell priming (59). GRHL3 (*epidermal development*), which encodes an epidermal differentiation-promoting transcription factor essential for skin barrier function, has been shown to suppress the expression of alarmins and proinflammatory genes during immune-mediated epidermal hyperplasia (60). Finally, the MAGE genes (*tumor antigens*) encode cancer-testis antigens (CTAs) that are physiologically expressed only in male germ cells but aberrantly expressed in various malignant tissues (61). These proteins are considered ideal immunotherapeutic targets in cancer due to their highly immunogenic nature and their restricted expression in normal tissues (61). MAGE-A proteins, including three of the four genes identified in our analysis (MAGEA2, MAGEA3, and MAGEA6), have recently been implicated in resistance to CTLA-4 ICB in melanoma patients (62). In that report, MAGE-A gene and protein overexpression in tumors was observed in ICB non-responders, and a negative correlation was observed between MAGE-A protein expression and key activators of autophagy with roles in antigen-specific T cell priming and stimulation of immunogenic cell death (62).

The findings from our mutual exclusivity studies support the possibility that in solid tumors, two predominant gene cassettes may functionally converge on the T cell-depleted tumor phenotype. To further examine this possibility, we tested these gene cassettes for clinical associations with immunotherapy response. Using RNAseq data from two cohorts of patients treated with nivolumab (Ipi Naïve and Ipi Prog), we found that a mean-based signature of the cluster 2 (C2) gene cassette was significantly inversely associated with nivolumab response in both pre-treatment and on-treatment tumor biopsies, as well as inversely associated with overall survival of patients in both cohorts. These findings indicate a potential clinical value for the C2 signature as a treatment-predictive biomarker. How the predictive power of the C2 signature compares to other markers of immunotherapy response warrants further investigation in larger treatment cohorts.

The non-random overrepresentation of CulPRIT genes within the 99th percentiles of many cancer types may reflect a selective advantage for their transcriptional upregulation that equates with immune evasion. Interestingly, several of our most consistently top-ranked CulPRITs have known functional roles in immune regulation. RCOR2 was ranked in the 99th percentile (by LFC method) in 12 of 23 tumor types. RCOR2 encodes a nuclear transcriptional corepressor that promotes the epigenetic silencing of gene transcription in neural stem cells (37) and suppresses the production of proinflammatory cytokines in a mouse model of aging (63). Moreover, RCOR2 binds and activates LSD1, a histone lysine demethylase recently identified as a potent inhibitor of anti-tumor immunity (37, 38). In the latter study, LSD1 ablation in a mouse melanoma model activated endogenous retrovirus (ERV) expression and type I IFN signaling, which stimulated the potent induction of T cell infiltration into tumors and enhanced ICB efficacy (38). Our finding that shRNA repression of RCOR2 leads to the induction of ERV, IFN, and ISG expression supports the hypothesis that RCOR2 expression phenocopies the T cell-excluded state induced by LSD1. Thus, that RCOR2 was discovered as a top CulPRIT may indicate that the transcriptional upregulation of RCOR2 in tumors confers a selective advantage via LSD1-mediated immune evasion—a mechanism with likely relevance to many cancer types according to our findings.

Interestingly, another top-ranking candidate in our study, KDM5B, also encodes a histone lysine demethylase, not unlike that of LSD1. KDM5B has been reported to suppress STING expression in breast cancer cell lines, thus short-circuiting the cGAS–STING–TBK1–IRF3 signaling axis important for innate immunity (64). Inhibition of KDM5B has also been shown to increase the expression of ISGs and potentiate resistance to infection by both DNA and RNA viruses (64). The CMTM4 gene ranked in the 99th percentile (by SC method) in 9 of 23 tumor types. CMTM4 and its homolog CMTM6 encode transmembrane proteins that positively regulate the PD-L1 protein pool in human tumor cells and dendritic cells (65). CMTM6, which has been functionally characterized to a greater extent than CMTM4, promotes PD-L1 stability through a direct binding interaction that reduces PD-L1 ubiquitination, thereby increasing its half-life (65). It has been suggested that such a stabilizing effect could occur in the immunological synapse between PD-1+ CD8+ T cells and PD-L1+ APCs, short-circuiting T cell priming (66).

Our *in vivo* confirmation of BMP7 as a negative regulator of intratumoral T cell abundance exemplifies the utility of our CulPRIT discovery platform for identifying candidate regulators of T cell trafficking in tumors for downstream functional analysis. *In vivo*, the expression of BMP7 in murine breast and colon cancer models led to the significant decrease in abundance of tumor-infiltrating CD8+ T cells. Indeed, BMP7 expression in the MC38 model also limited T cell infiltration characteristically induced by effective ICB treatment. However, in this model, BMP7 expression synergized with ICB treatment to suppress tumor growth. This observation may be explained, in part, by the fact that BMPs can function in cancer as tumor suppressors depending on the cancer type and context (44). Results from our scRNA-seq studies support the possibility that a BMP7-mediated repression of TGFB1 expression in multiple cell types within the TME may function to alleviate immunosuppression, and thus, enhance ICB effectiveness. Nevertheless, our findings support roles for BMP7 in both T cell exclusion and ICB-mediated suppression of MC38 tumor growth.

Several limitations of this study are notable. First, not all CulPRITs identified are expected to functionally contribute to the T cell-cold tumor state. Some could merely reflect passenger genes expressed within larger transcriptional networks without direct functional consequences. Second, our analyses did not seek to discern the cellular origin of CulPRITs, whose mRNA transcription could originate in either cancer cells or non-malignant stromal cells within the TME. Third, it is also possible that a fraction of our CulPRITs are genes expressed at steady-state levels in immune-cold tumors but transcriptionally repressed by leukocyte-derived cytokines overexpressed in immune-hot tumors. Further biological characterization will be necessary to establish the immune modulatory functions of the genes identified in this study.

## Conclusion

This work demonstrates that immunologically cold tumors of diverse histologic origin share more transcriptomic similarities than previously recognized. Genes and gene subsets identified in this study may function to facilitate tumor immune evasion, as evidenced by confirmatory phenotypic observations associated with RCOR2 and BMP7 expression. In addition, CulPRIT gene subsets, such as the C2 gene signature, are predictive of immunotherapy response and survival of melanoma patients. Further understanding of how these genes and their associated pathways function in immune regulation could reveal novel, tumor-agnostic immunotherapeutic targets with broad translational potential.

## Data Availability Statement

Plasmids developed in this study are available through Addgene. Sequence data are publicly available through Firebrowse.org (Broad Institute of MIT and Harvard) and in accordance with the TCGA data usage policy (https://www.cancer.gov/about-nci/organization/ccg/research/structural-genomics/tcga/history/policies). Other raw data supporting the conclusions of this article will be made available by the authors, without undue reservation, to any qualified researcher.

## Ethics Statement

The animal study was reviewed and approved by Wake Forest School of Medicine Institutional Animal Care and Use Committee.

## Author Contributions

ER and LM conceived of and designed the study, and wrote the manuscript. ER, JC, and JWC performed the bioinformatics analyses. RD'A provided statistical support. ER conducted wet lab experiments. AP assisted with *in vivo* experiments and conception of cloning strategies. KS, HW, and YL provided animal model guidance. CP, WZ, GJ, and YL contributed to data interpretation. All authors contributed to aspects of the data analysis or data interpretation, the writing of the manuscript, and approved the final manuscript.

### Conflict of Interest

The authors declare that the research was conducted in the absence of any commercial or financial relationships that could be construed as a potential conflict of interest.

## Supplementary Material

Supplementary Datasheet 1 A series of tables detailing genetic data.

## Non-standard Abbreviations

CulPRITs: candidate protein regulators of immune trafficking
EBP: exact binomial probability
MPR: median percentile rank
Nivo: nivolumab
Ipi: ipilimumab.
