# Supplementary material for: Transcriptomic Features of T Cell-Barren Tumors Are Conserved Across Diverse Tumor Types
Source: Front Immunol. 2020 Feb 13;11:57. doi: 10.3389/fimmu.2020.00057 (PMC7031496; doi:10.3389/fimmu.2020.00057)
Supplement: Supplementary file 2 [file Data_Sheet_2.pdf]

Supplementary Material

Supplementary Figure 1. T^{SIG} and C^{SIG} correlation analysis with T cell immune gene signatures.

Supplementary Figure 2. T^{SIG} and C^{SIG} tend to associate with favorable survival outcomes in diverse tumors.

Supplementary Figure 3. T^{SIG} quantitation of intratumoral CTL abundance reflects pathohistological classification of TIL.

Supplementary Figure 4. Genes associated with T cell exclusion are shared across diverse cancer types.

Supplementary Figure 5. Mutual exclusivity analysis of MPR-CulPRITs, with genes identified.

Supplementary Figure 6. Top ranking CulPRITs are enriched in the top percentile rank of diverse cancer types.

Supplementary Figure 7. BMP7 is highly expressed in immunologically privileged tissues.

Supplementary Figure 8. BMP7-expressing 4T1-S and MC38 stable cell lines secrete mature protein.

Supplementary Figure 9. Generation of stable murine cancer cell lines that inducibly/constitutively express BMP7.

Supplementary Figure 10. 4T1-S tumors exhibit increased CTL infiltrate over time relative to parental 4T1.

Supplementary Figure 11. 4T1-S-pR26 Ctl versus 4T1-S-pR26 BMP7 tumors display no differences in growth kinetics.

Supplementary Figure 12. BMP7-dependent tumor growth characteristics in the MC38 colon adenocarcinoma model.

Supplementary Table 1. Summary of gene composition of T cell gene signatures used in correlation analysis.

Supplementary Table 2. Top-ranking CulPRITs possess documented immunomodulatory functionality.

Supplementary Table 3. Exact binomial probabilities associated with the discovery and ranking of CulPRITs (C^{SIG}-based).

Supplementary Table 4. Mutually exclusive CulPRIT gene expression patterns are consistently negatively correlated across tumor types.

Supplementary Table 5. qPCR primers used to examine IFNs, ISGs and HERVs in MCF7 cells expressing shRNA targeting RCOR2.

Supplementary Methods

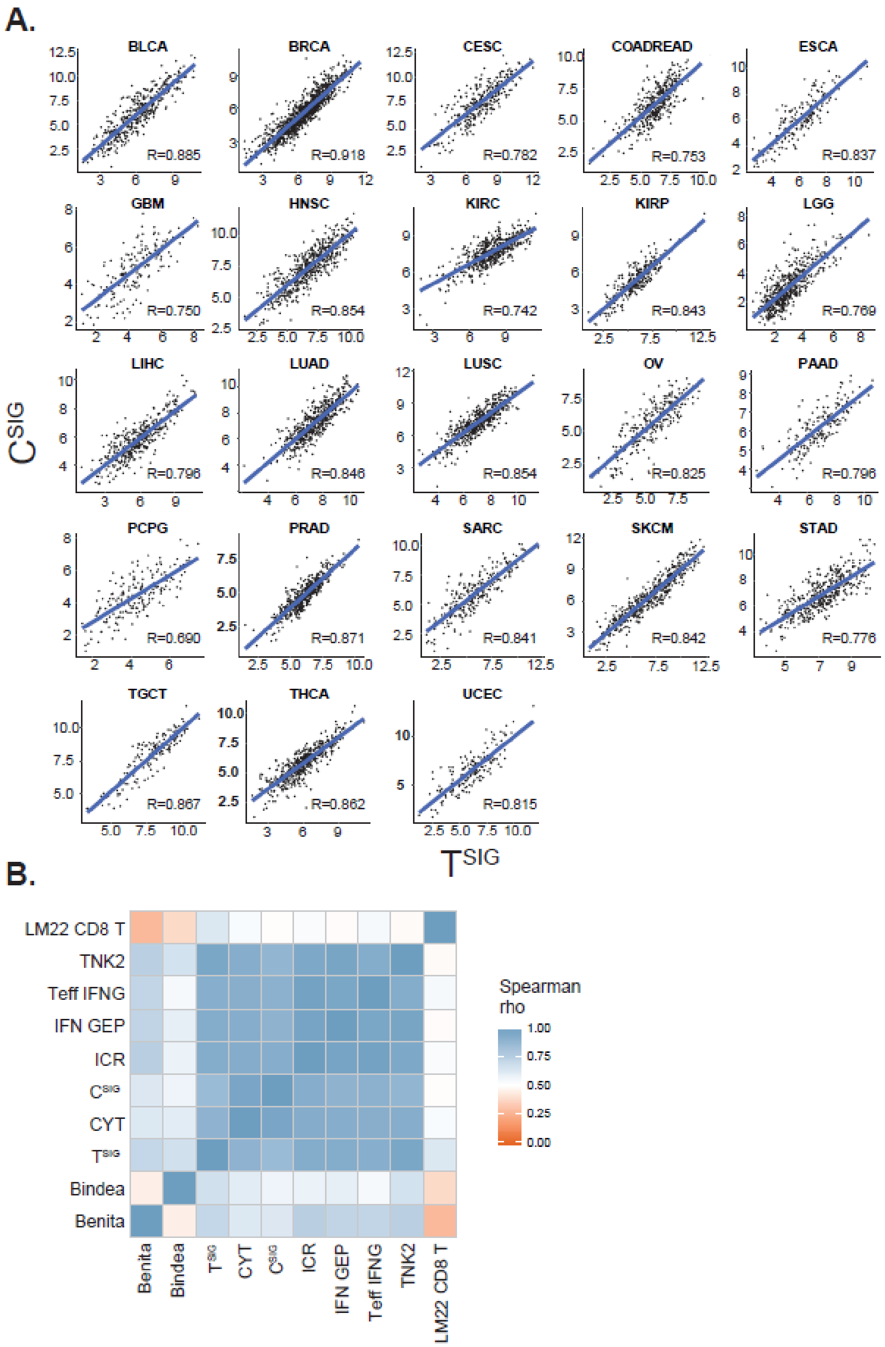

Supplementary Figure 1. T^{SIG} and C^{SIG} correlation analysis with T cell immune gene signatures. (A) Shown is the Spearman correlation between T^{SIG} and C^{SIG} scores across the 23 tumor types analyzed in this study ($p < 0.0001$ for all). **(B)** Using a pan-tumor dataset of harmonized TCGA RNAseq data sets (9,391 tumors; NCI Genomic Data Commons, GDC; https://portal.gdc.cancer.gov), T^{SIG} and C^{SIG} metagene scores were computed and compared by Spearman's rank correlation to published immune gene signatures that reflect T cell biology (see **Supp. Table 1** for summary and references) (Benita et al., 2010; Bindea et al., 2013; Rooney et al., 2015; Chifman et al., 2016; Fehrenbacher et al., 2016; Ayers et al., 2017; Bertucci et al., 2018; Thorsson et al., 2018).

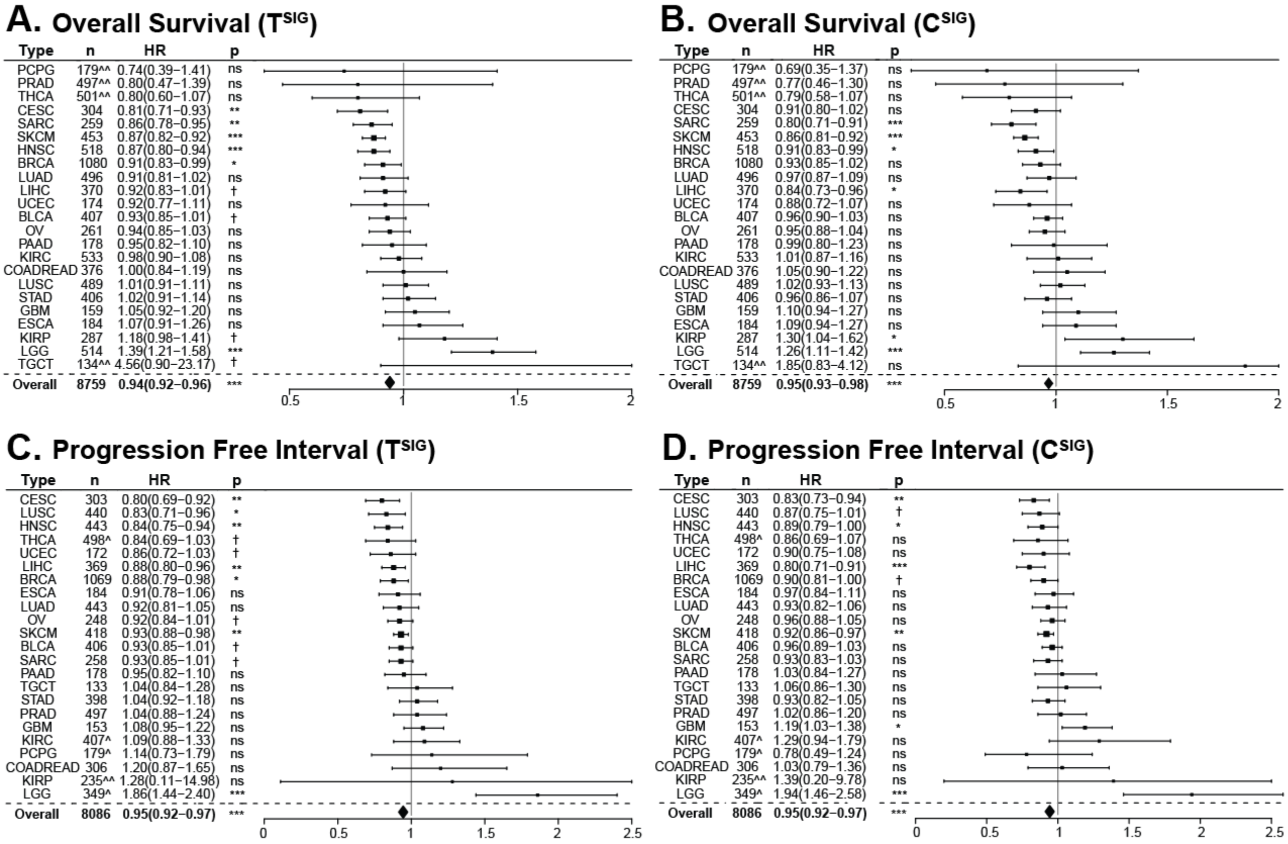

Supplementary Figure 2. T^{SIG} and C^{SIG} tend to associate with favorable survival outcomes in diverse tumors. Multivariable cox proportional hazards models included patient age at diagnosis (continuous), race (categorical), gender (categorical), cancer stage (categorical) and T^{SIG} or C^{SIG} scores (continuous). Model endpoints included **(A, B)** overall survival and **(C, D)** progression free interval (Liu et al., 2018; Thorsson et al., 2018) for T^{SIG} and C^{SIG}, respectively. n, number of evaluable patients. HR, Hazard Ratio. p, p-value. Significance codes: †, $p < 0.1$; *****, $p < 0.05$; ******, $p < 0.01$; and *******, $p < 0.001$. ^, less than 10% of patients had events; ^^, less than 5% of patients had events. The R package "survival" (Therneau, 2015) was used for multivariable analysis to compute hazard ratios, confidence intervals and p-values. The R package "forestplot" (Max Gordon, 2017) was used for data visualization.

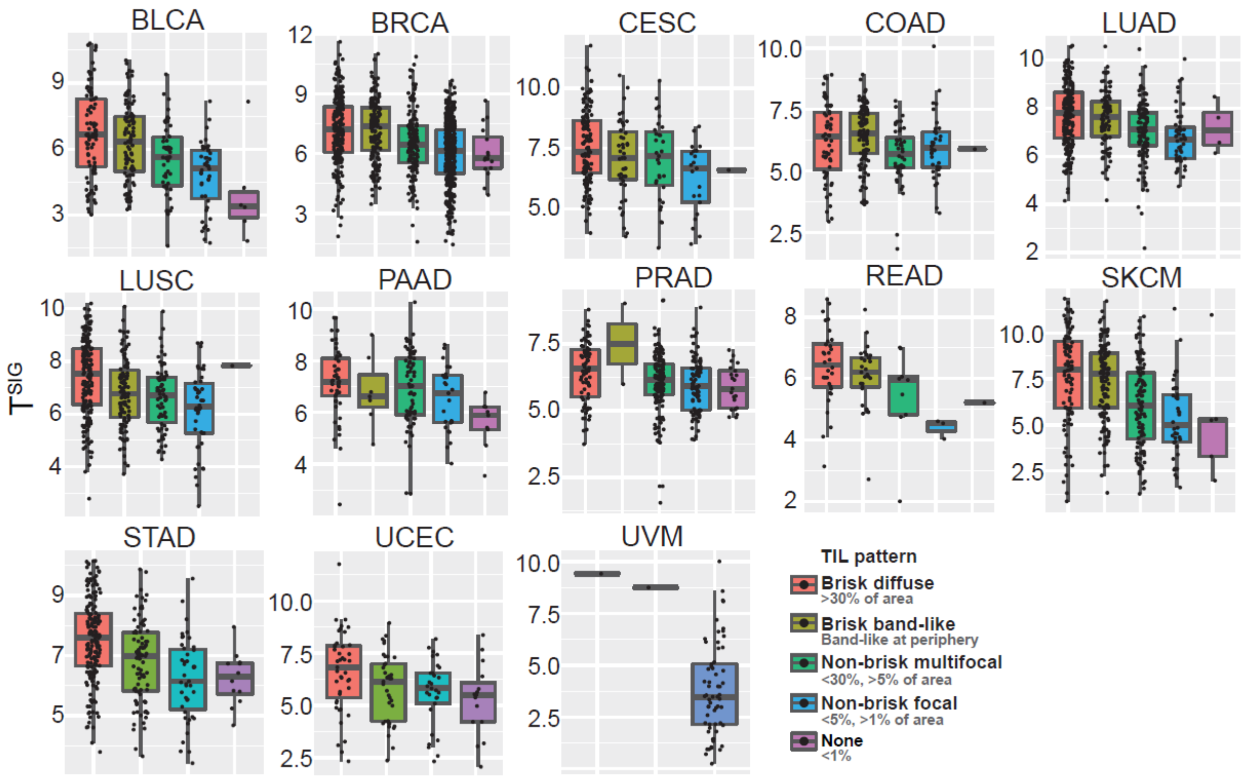

Supplementary Figure 3. T^{SIG} quantitation of intratumoral CTL abundance reflects pathohistological classification of TIL. Shown are box and whisker plots of the distributions of T^{SIG} metagene scores (y-axes) corresponding to TCGA tumors that were previously classified to TIL pathohistological categories (brisk diffuse, brisk band-like, non-brisk multifocal, non-brisk focal, and none) as reported in (Saltz et al., 2018).

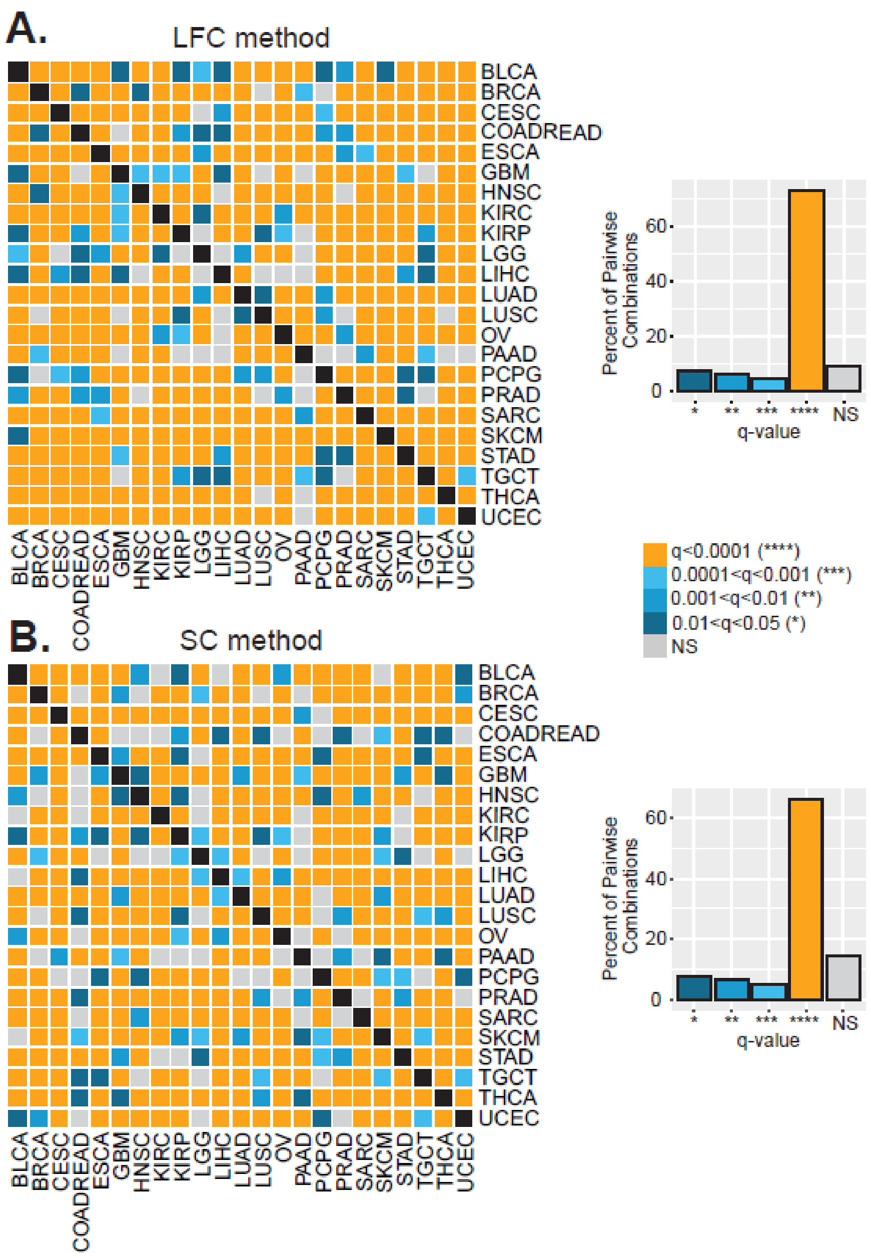

Supplementary Figure 4. Genes associated with T cell exclusion are shared across diverse cancer types. Genes comprising the top (99th) percentile rank for each of 23 tumor types, ranked using C^{SIG} scores, were compared by Chi-squared test for all pairwise tumor group combinations. Heatmaps show the significance (see color key) of overlapping 99th percentile genes for each tumor group pairwise combination, where gene ranking was based on **(A)** the LFC method, or **(B)** the SC method. Bar charts display the percent of pairwise comparisons that achieved statistical significance (q, FDR-corrected p values) at indicated thresholds for each method.

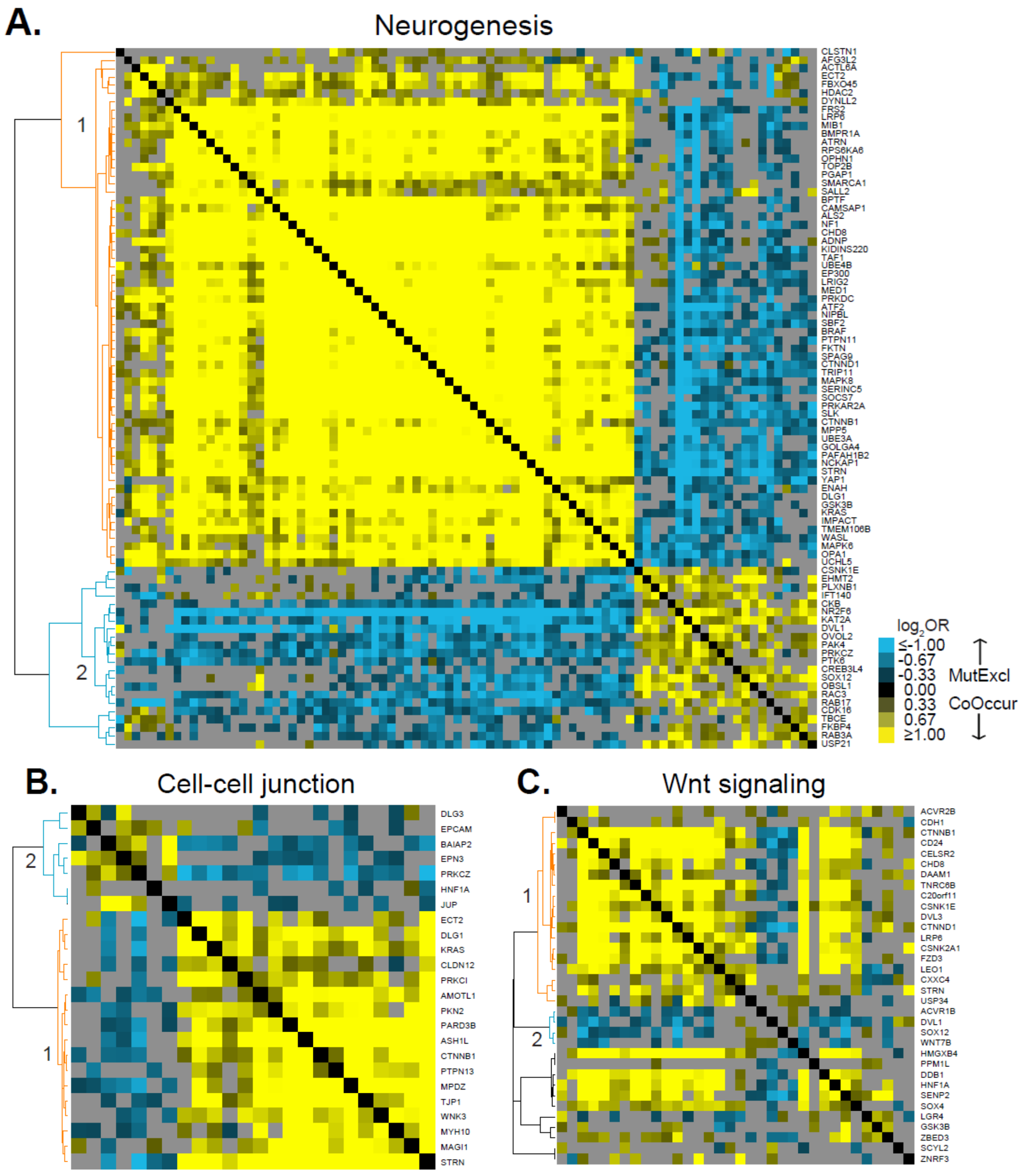

Supplementary Figure 5. Mutual exclusivity analysis of MPR-CulPRITs, with genes identified. We conducted pan-tumor correlation studies of median percentile rank (MDR)-defined CulPRIT genes (n = 1,417) in CD8-Low tumors. Pan-tumor analysis of gene mutual exclusivity or co-occurrence was performed on CulPRIT gene subsets. Shown are clustered heatmaps of gene-pair $\log_2$ odds ratios (OR) derived from Fisher's exact test analysis. Blue denotes negative associations (mutual exclusion); yellow depicts positive associations (co-occurrence; see color key). Gray indicates sub-significant associations ($q > 0.001$). Genes comprising the major dendrogram clusters are indicated by cluster 1 (orange branch) and cluster 2 (blue branch). Shown are heatmaps corresponding to the subsets of **(A)** neurogenesis-annotated genes, **(B)** cell-cell junction-annotated genes, and **(C)** Wnt signaling-annotated genes.

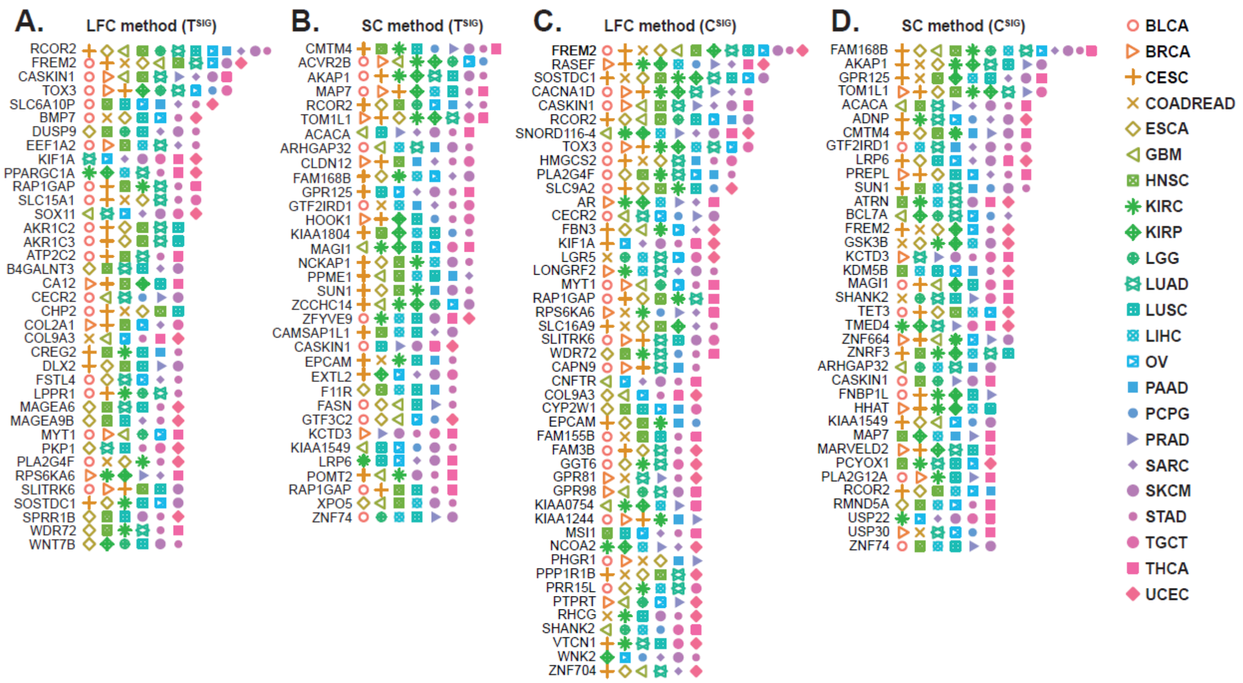

Supplementary Figure 6. Top ranking CulPRITs are enriched in the top percentile rank of diverse cancer types. CulPRIT genes identified by exact binomial probability (EBP) are shown. Genes that fell into the top (99th) percentile rank 6 or more times out of 23 by **(A)** the LFC method and **(B)** the SC method are displayed relative to tumor types. For a complete list of significant genes identified based on the exact binomial probability statistic (e.g., including those identified in the 99th percentile 5 or more times) refer to Supp. File 1.

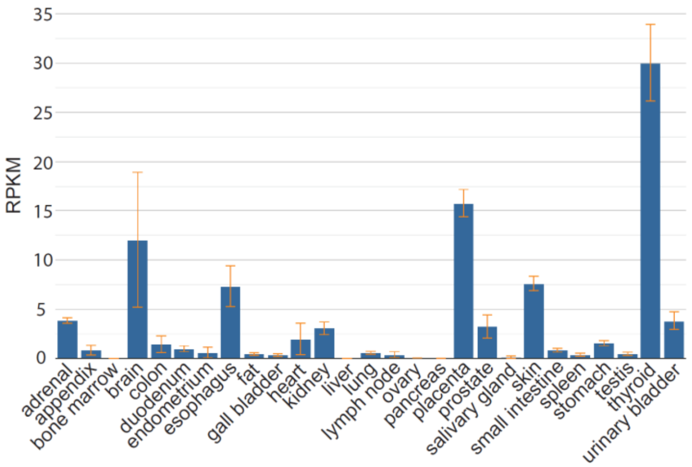

Supplementary Figure 7. BMP7 is highly expressed in immunologically privileged tissues such as the brain and placenta.
(NCBI Gene database: https://www.ncbi.nlm.nih.gov/gene/655)

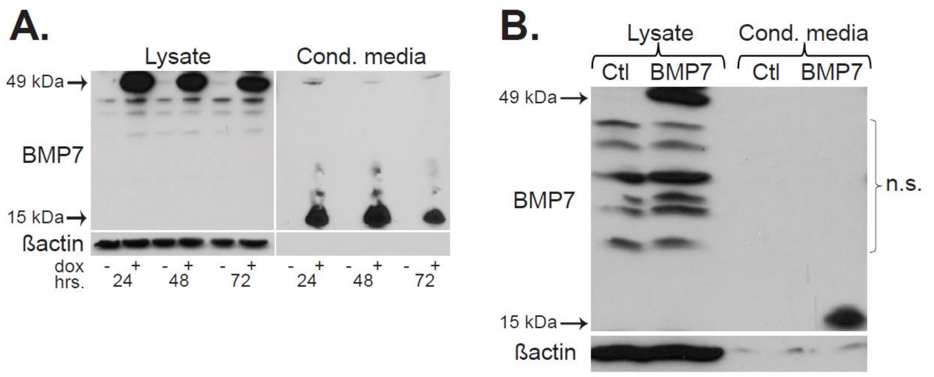

Supplementary Figure 8. BMP7-expressing 4T1-S and MC38 stable cell lines secrete mature protein. Western blots of BMP7-expressing **(A)** 4T1-S and **(B)** MC38 cells confirming that stable cell lines secrete mature protein. Antibodies used for western blot were: rabbit-anti-BMP7 (Abcam #ab27569, 1:2,000), goat-anti-rabbit IgG-HRP (Santa Cruz Biotechnology, #sc-2004, 1:5,000), mouse-anti-βactin (Sigma #A5441, 1:10,000), and goat-anti-mouse IgG-HRP (Santa Cruz Biotechnology, #sc-2005, 1:10,000).

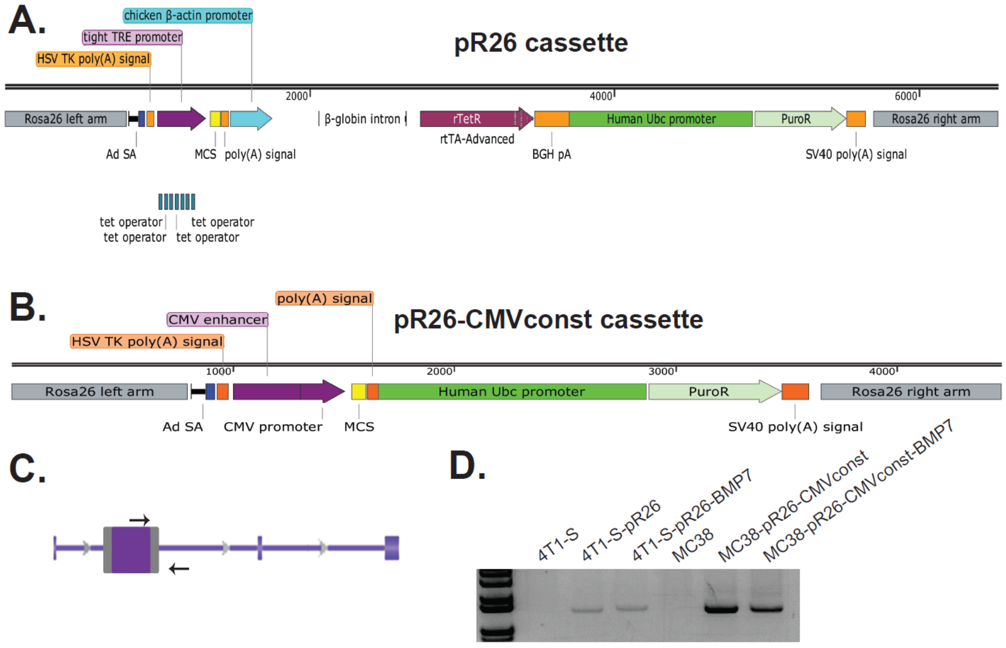

Supplementary Figure 9. Generation of stable BMP7-expressing murine cancer cell lines. (A) Schematic of pR26 inducible expression cassette. **(B)** Schematic of pR26-CMVconst constitutive expression cassette. **(C)** Depiction of genomic insertion of pR26/pR26-CMVconst cassettes into the first intron of the murine ROSA26 locus; arrows depict genomic DNA PCR primers used for validation of stable cell lines, as shown in (D). **(D)** PCR amplification of 1135 bp DNA fragment confirming stable integration of expression cassettes in 4T1-S/MC38 cells; labels represent source of genomic DNA input.

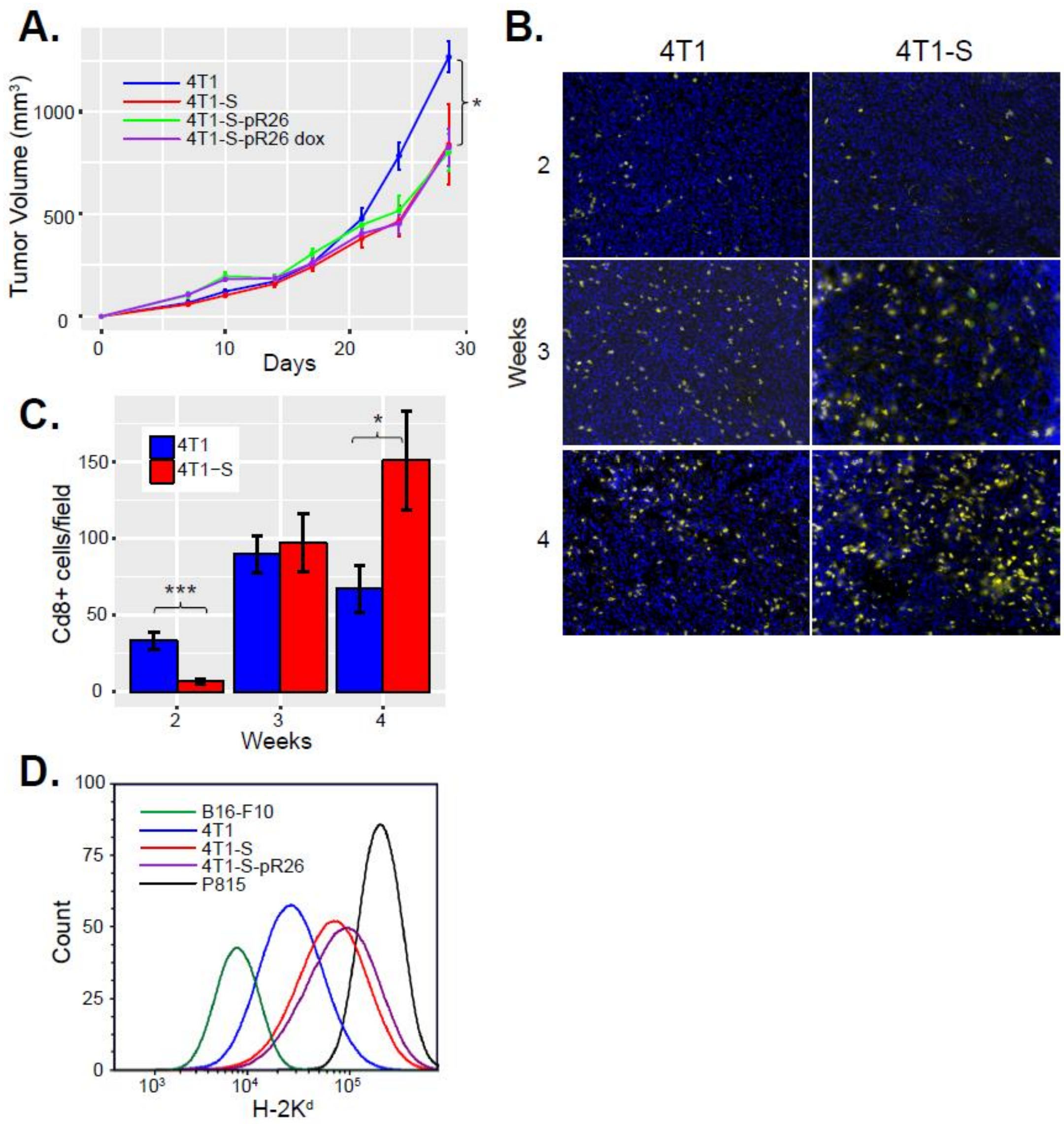

Supplementary Figure 10. 4T1-S cells exhibit increased CTL infiltrate over time relative to parental 4T1. (A) 4T1-S cell lines exhibit slower in vivo tumor growth kinetics relative to parental 4T1 tumors ($p < 0.05$; single factor ANOVA). **(B)** Representative immunofluorescent images of tumors harvested at 2, 3 and 4 wks post tumor cell implantation; CD8a (yellow) and DAPI (blue). **(C)** Quantitation of CD8a+ CTL abundance over time as represented in panel (B); *, $p < 0.05$; ***, $p <0 .001$ (Student's t-test). n = 5 mice per group per time point. Error bars show the standard error of the mean (panels A and C). **(D)** Flow cytometry analysis of H-2K^d MHC class I surface expression shows elevated levels in 4T1-S cells lines relative to parental 4T1 cells.

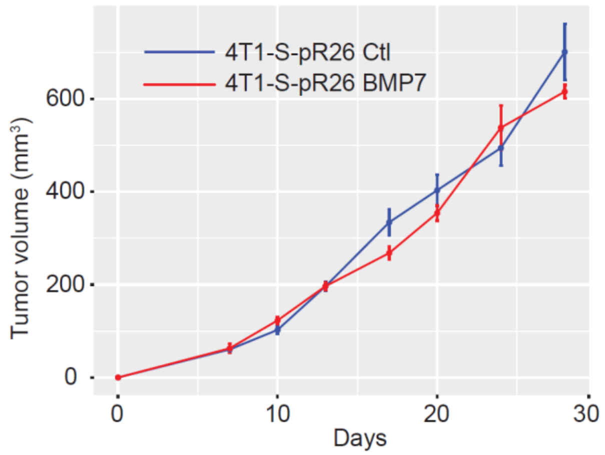

Supplementary Figure 11. 4T1-S-pR26 Ctl versus 4T1-S-pR26 BMP7 tumors display no differences in growth kinetics. n = 5 mice per group. Error bars show the standard error of the mean.

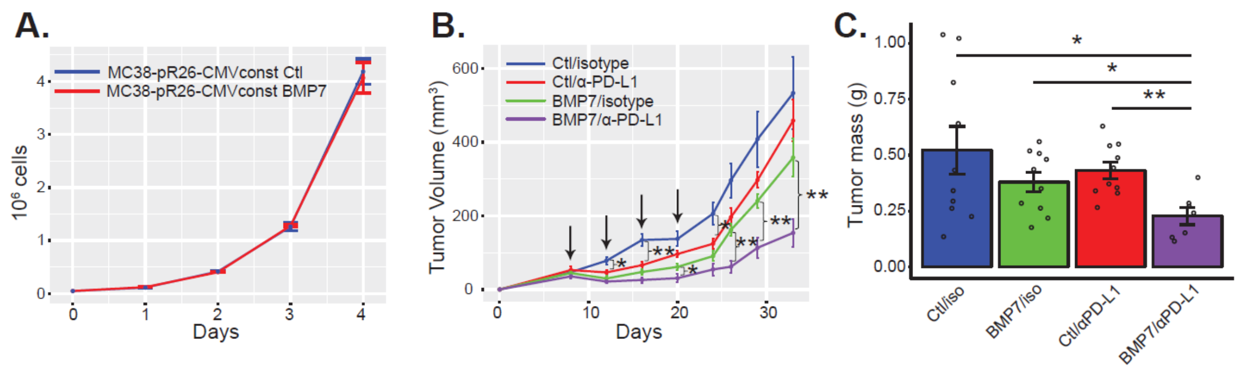

Supplementary Figure 12. BMP7-dependent tumor growth characteristics in the MC38 colon adenocarcinoma model. (A) Constitutive BMP7-expressing MC38 cell lines display no differences in in vitro growth rates over the course of a 96-hour proliferation assay (cell growth assayed in triplicate). **(B)** Tumor growth kinetics of control versus BMP7-expressing MC38 colon tumors treated with or without anti-PD-L1 checkpoint antibodies show a BMP7-dependent growth rate decrease over time that is further decreased upon anti-PD-L1 antibody treatment. Black arrows represent intraperitoneal anti-PD-L1 antibody injections (10 mg/kg). **(C)** Depiction of tumor mass as assessed at 5 weeks post tumor cell inoculation; *, $p < 0.05$; **, $p < 0.01$ (Student's t-test). n = 10 mice per group. Error bars show the standard error of the mean.

Supplementary Table 1. Summary of the gene composition of T cell gene signatures used in correlation analysis (see Supp. Fig. 1B).

Signature	Description	Genes (No.)	PMID
**LM22 CD8 T**	$CD8^+$ T cell-enriched genes	68	29628290
**TNK2**	T cell/NK cell-enriched genes	33	27871313
**Teff IFNG**	Associated with activated T cells, immune cytolytic activity, and interferon-γ expression	8	26970723
**IFN GEP**	IFN-γ-responsive genes related to antigen presentation, chemokine expression, cytotoxic activity, and adaptive immune resistance	18	28650338
**ICR**	Genes involved in Th-1 and interferon signalling, Th-1 chemoattraction, and cytotoxic functions	20	30353048
**C^{sig}**	Canonical CTL cytotoxic effector molecules	4	This study
**CYT**	Canonical CTL cytotoxic effector molecules	2	25594174
**T^{sig}**	Canonical CTL surface markers	4	This study
**Bindea**	CD8+ T cell-related genes	36	24138885
**Benita**	T cell-enriched genes	32	20410506

Supplementary Table 2. Top-ranking CulPRITs possess documented immunomodulatory functionality.

Top CulPRIT	Immunomodulatory functionality	PMID
**BMP7**	• TGF-β superfamily member<br>• Promotes M2 macrophage polarization<br>• Antagonizes pro-inflammatory cytokine production and promotes anti-inflammatory cytokine production<br>• Regulation of monocyte adhesion/migration	11319750; 22720873; 27669124
**CMTM4**	• Stabilizes PD-L1 protein	28813410
**DUSP9**	• Modulates immune effector signaling responses	17114416
**GTF2IRD1**	• Represses adipose inflammation<br>• Reduces proinflammatory signaling	29320702
**KDM5B**	• Immune repression via suppression of STING	30080846
**LRP5**	• Novel marker of anti-inflammatory macrophages	26739212; 25656427
**RCOR2**	• Involved in an inflammatory suppressive program<br>• LSD1 binding partner<br>• Ablation of LSD1 potentiates immunotherapy	25051986 29937226
**TOX3**	• Transcription factor related to TOX, which is required for T cell development	18195075; 26556952; 27080130

Supplementary Table 3. Exact binomial probabilities associated with the discovery and ranking of CulPRITs. The exact binomial probability statistic was applied to determine the probability of any gene showing up in the top percentile rank k or more out of 23 times. The number and identity of genes derived using the C^{SIG} signature (for both LFC and SC methods) that are found at each of these thresholds are shown.

k out of 23*	Bonferroni p**	LFC (No. genes)	Genes	SC (No. genes)	Genes
**13**	2.12×10^{-16}	1	*FREM2*	1	*FAM168B*
**12**	2.48×10^{-14}	0	-	-	-
**11**	2.46×10^{-12}	0	-	-	-
**10**	2.06×10^{-10}	2	*RASEF, SOSTDC1*	-	-
**9**	1.46×10^{-8}	6	*CACNA1D, CASKIN1, RCOR2, SNORD116-4, TOX3*	3	*AKAP1, GPR125, TOM1L1*
**8**	8.65×10^{-7}	3	*HMGCS2, PLA2G4F, SLC9A2*	7	*ACACA, ADNP, CMTM4, GTF2IRD1, LRP6, PREPL, SUN1*
**7**	4.28×10^{-5}	12	*AR, CECR2, FBN3, KIF1A, LGR5, LONRF2, MYT1, RAP1GAP, RPS6KA6, SLC16A9, SLITRK6, WDR72*	12	*ATRN, BCL7A, FREM2, GSK3B, KCTD3, KDM5B, MAGI1, SHANK2, TET3, TMED4, ZNF664, ZNRF3*
**6**	1.74×10^{-3}	23	*CAPN9, CNTFR, COL9A3, CYP2W1, EPCAM, FAM155B, FAM3B, GGT6, GPR81, GPR98, KIAA0754, KIAA1244, MSI1, NCOA2, PHGR1, PPP1R1B, PRR15L, PTPRT, RHCG, SHANK2, VTCN1, WNK2, ZNF704*	14	*ARHGAP32, CASKIN1, FNBP1L, HHAT, KIAA1549, MAP7, MARVELD2, PCYOX1, PLA2G12A, RCOR2, RMND5A, USP22, USP30, ZNF74*
**5**	5.76×10^{-2}	64	*ABCC8, AGR2, BCAS1, BMP7, C9orf152, CACNA2D2, CAPN13, CAPN6, CAPN8, CDKL5, CEACAM5, CHP2, CLDN3, CNTN3, COL2A1, COL4A6, CRABP1, DCX, DDI2, DKFZp686O24166, DUSP9, EEF1A2, ERBB4, FADS6, GJB1, GPC3, GSTA1, HS6ST3, IGSF9, ILDR2, IYD, KIAA1549, KLHL23, KSR2, LPPR1, MAPK4, MUM1L1, NKAIN1, OCLN, PALM3, PCDHGB1, PCSK2, PNCK, PRAP1, PRKG2, PTPLB, RAB3B, ROBO2, SBK1, SLC6A10P, SLC7A4, SLC9A4, SOX11, SOX21, SYT13, SYTL5, TMEM38A, TMPRSS2, UGT1A10, UGT1A6, UGT1A9, UPK1B, USH1C, VWA2*	53	*ACVR2B, ANKRD46, ARID2, ATP8B1, ATP9A, BPTF, CACNA1D, CDH1, CECR2, CUL4A, DNAL1, EPB41L5, GPR56, HOOK1, ICA1, IFT140, KIAA1543, KIAA1737, KLHL23, LRBA, LRP5, MAP4K3, MFN1, MIPOL1, MYEF2, MYST2, MYST4, NCKAP1, NCOA6, OCLN, OPHN1, PLEKHH1, PLXNB1, RALGPS1, RAP1GAP, SALL2, SBK1, SH3RF1, SLC25A13, SMARCC1, SOCS7, SPATA6, TMTC4, TRIM45, TTC3, TUB, USP46, ZCCHC14, ZKSCAN2, ZMYND11, ZNF462, ZNF704, ZNF774*

* k is the number of times that a gene falls into the top percentile rank out of 23 tumor types.
** Represents the likelihood of any one gene falling into the top percentile rank k out of 23 times.

Supplementary Table 4. Mutually exclusive CulPRIT gene expression patterns are consistently negatively correlated across tumor types. Metagenes representing clusters 1 and 2 (C1 and C2; clusters represent genes that predominantly display patterns of mutual exclusivity or co-occurrence; refer to Fig. 4 and 6) were calculated (by geometric mean) from T^{SIG}-low (low tertile) tumors for each cancer type. Spearman correlation between the two metagenes was performed for each tumor type to test for associations. Consistent negative correlation between these clusters across cancer types suggests that there is not a cancer-type bias being introduced into the pan-cancer analysis that concluded that genes comprising these categories exhibited patterns of mutual exclusivity or co-occurrence.

		Fig. 4B (CulPRITs; LFC/SC median-rank-prioritized) C1 vs C2		Fig. 4C (Neurogenesis) C1 vs C2		Fig. 4D (Cell-cell junction) C1 vs C2		Fig. 4E (Wnt signaling) C1 vs C2		Fig. 6B (CulPRITs; Exact Binomial-prioritized) C1 vs C2	
**Type**	**n**	**R**	**p**	**R**	**p**	**R**	**p**	**R**	**p**	**R**	**p**
BLCA	136	-0.58	9.43E-14	-0.48	4.69E-09	-0.30	3.24E-04	-0.06	4.75E-01	0.15	8.91E-02
BRCA	360	-0.62	5.07E-40	-0.55	2.90E-30	-0.40	4.66E-15	-0.19	3.28E-04	-0.18	7.42E-04
CESC	101	-0.27	6.78E-03	-0.23	2.00E-02	-0.24	1.68E-02	0.13	1.90E-01	-0.23	2.18E-02
COADREAD	126	-0.42	1.03E-06	-0.40	3.87E-06	-0.26	3.57E-03	0.00	9.83E-01	-0.07	4.05E-01
ESCA	61	-0.28	2.82E-02	-0.31	1.48E-02	-0.24	6.70E-02	0.38	2.79E-03	0.10	4.41E-01
GBM	51	-0.24	7.45E-02	-0.14	3.29E-01	-0.21	1.33E-01	0.34	1.21E-02	0.36	8.05E-03
HNSC	174	-0.32	1.42E-05	-0.18	2.07E-02	-0.26	5.39E-04	0.21	5.70E-03	0.19	1.06E-02
KIRC	178	-0.55	1.62E-15	-0.55	2.36E-15	-0.25	9.69E-04	-0.13	9.01E-02	-0.17	2.07E-02
KIRP	97	-0.63	5.80E-12	-0.58	7.65E-10	-0.27	6.75E-03	-0.22	2.95E-02	0.00	9.94E-01
LGG	177	-0.60	8.24E-19	-0.57	1.79E-16	-0.14	6.86E-02	-0.06	4.51E-01	-0.03	6.98E-01
LIHC	124	-0.35	8.21E-05	-0.20	3.05E-02	-0.07	4.28E-01	0.16	8.49E-02	0.09	3.02E-01
LUAD	172	-0.31	3.29E-05	-0.32	1.56E-05	-0.28	2.56E-04	0.07	3.43E-01	0.14	6.20E-02
LUSC	167	-0.21	5.98E-03	-0.21	7.42E-03	-0.24	1.73E-03	0.28	3.20E-04	0.08	3.34E-01
OV	88	0.03	7.89E-01	0.03	7.85E-01	-0.03	7.60E-01	0.41	9.96E-05	0.22	3.68E-02
PAAD	60	-0.61	3.68E-07	-0.61	2.66E-07	-0.03	7.98E-01	-0.19	1.56E-01	-0.14	2.98E-01
PCPG	61	-0.54	7.23E-06	-0.46	2.13E-04	-0.02	9.07E-01	-0.18	1.67E-01	-0.21	1.14E-01
PRAD	166	-0.58	1.59E-16	-0.58	3.67E-16	-0.46	5.80E-10	0.11	1.70E-01	-0.17	2.76E-02
SARC	88	-0.28	8.74E-03	-0.10	3.34E-01	-0.20	6.08E-02	-0.07	5.00E-01	0.13	2.28E-01
SKCM	157	-0.61	2.38E-17	-0.60	2.42E-16	-0.21	7.57E-03	-0.04	6.03E-01	0.18	2.89E-02
STAD	138	-0.32	1.17E-04	-0.28	8.78E-04	-0.13	1.21E-01	0.04	6.64E-01	0.13	1.29E-01
TGCT	52	-0.51	1.39E-04	-0.37	7.32E-03	-0.23	1.00E-01	0.41	2.69E-03	0.03	8.43E-01
THCA	170	-0.68	3.05E-24	-0.67	1.67E-23	-0.06	4.59E-01	-0.14	6.29E-02	-0.27	3.17E-04
UCEC	59	-0.51	4.50E-05	-0.36	5.10E-03	-0.45	4.45E-04	0.00	9.77E-01	0.24	6.56E-02

Supplementary Table 5. qPCR primers used to examine IFNs, ISGs and HERVs in MCF7 cells expressing shRNA targeting RCOR2.

Target genes	Primer name	Sequence
human GAPDH	h-GAPDH-qPCR-F1<br>h-GAPDH-qPCR-R1	AACGGGAAGCTTGTCATCAA<br>TGGACTCCACGACGTACTCA
human IFN-α	h-IFNα-qPCR-F<br>h-IFNα-qPCR-R	GGAGGTTGTCAGAGCAGA<br>AATGACAGAATTCATGAAAGCGT
human IFN-β	h-IFNβ-qPCR-F<br>h-IFNβ-qPCR-R	GAAACTGAAGATCTCCTAGCCT<br>GCCATCAGTCACTTAAACAGC
human IL-28A	h-IL28A-qPCR-F<br>h-IL28A-qPCR-R	CAGCCTCAGAGTGTTTCTTCT<br>TCCAGTCACGGTCAGCA
human OASL	h-OASL-qPCR-F<br>h-OASL-qPCR-R	CCCATCACGGTCACCATTG<br>GCAGAAATTTCCAGGACCAC
human ISG15	h-ISG15-qPCR-F<br>h-ISG15-qPCR-R	CGAACTCATCTTTGCCAGTACA<br>CCTTCAGCTCTGACACC
human HERV-E	HERV-E-qPCR-F<br>HERV-E-qPCR-R	GGTGTCACTACTCAATACAC<br>GCAGCCTAGGTCTCTGG
human HERV-K	HERV-K-qPCR-F<br>HERV-K qPCR-R	ATTGGCAACACCGTATTCTGCT<br>CAGTCAAAATATGGACGGATGGT
human HML-2	HML-2-qPCR-F3<br>HML-2-qPCR-R3	AAAGAACCAGCCACCAGG<br>CAGTCTGAAAACTTTTCTCTA

Supplementary Methods

Construction of plasmids. *Cloning of pR26 construct for genomic alteration of the ROSA26 locus.* Genomic DNA from 4T1 cells was extracted and used as a template for PCR amplification of left and right ROSA26 homology arms. ROSA26 homology arm PCR products were blunt cloned into pCR-Blunt II-TOPO (Invitrogen) and sequence-verified (yielding pCRBl-R26 L and R, respectively). The left homology arm was subcloned into the KpnI site of pCRBl-R26 R (which had the XhoI site destroyed to facilitate downstream steps), yielding pCRBl-R26 L/R. Separately, duplexed oligos encoding an MCS were cloned into the XbaI site of the tetracycline-inducible pTetON7 plasmid (kindly gifted by Dr. Guangchao Sui, Wake Forest School of Medicine, Winston-Salem, NC), and a synthetic poly(A) (SPA) signal was introduced into the MluI site of the MCS. The modified pTetON7 plasmid was digested with ClaI/XhoI and the fragment encoding the tight TRE promoter, rtTA-Advanced transactivator, and PuroR gene was subcloned into the ClaI/XhoI-digested pCRBl-R26 L/R homology arm plasmid and sequence-verified (yielding pCRBl-R26). Primers were designed to amplify the following fragments to facilitate Gibson assembly: bGH poly(A) from pCDNA3.1(+) (Invitrogen); human Ubc promoter from pENTR 5' UbcP (Invitrogen); PuroR from pCRBl-R26; and the SV40 poly(A) signal from pCDNA3.1(+). The pCRBl-R26 plasmid was digested with XhoI/AscI, the large fragment was gel-purified and mixed with the above PCR products in a Gibson assembly reaction (New England

Biolabs), and the resultant construct was sequence-verified (pCRBl-R26-Gib). Finally, to ensure termination of endogenous ROSA26 transcripts a gene block encoding the adenovirus major late transcript splice acceptor and HSV TK poly(A) signal was cloned into the ClaI site of pCRBl-R26-Gib to yield the final construct, termed *pR26*, which was sequence-verified and deposited to Addgene (# 127372). All restriction enzymes were purchased from New England Biolabs, and all DNA oligomers and gene blocks were purchased from Integrated DNA Technologies.

Cloning of pR26-CMVconst construct for genomic alteration of the ROSA26 locus. To mediate constitutive candidate gene expression, the pR26 construct was modified. A restriction site-flanked (XhoI/AscI) gene block encoding the adenovirus major late transcript splice acceptor, HSV TK poly(A) signal, and CMV enhancer/promoter was cloned into the XhoI/AscI double-digested pR26 construct. The resultant construct, termed *pR26-CMVconst*, was sequence-verified and deposited to Addgene (# 127373).

Cloning of pR26-mBMP7 and pR26-CMVconst-mBMP7. Total RNA was harvested from 4T1 cells using Trizol (Invitrogen), and cDNA was synthesized using TaqMan reverse transcription reagents (ThermoFisher). Mouse BMP7 was PCR-amplified using 4T1 cDNA as a template, and cloned into the NheI/MluI sites of either pR26 or pR26-CMVconst. The resultant plasmids, termed *pR26-mBMP7* or *pR26-CMVconst-mBMP7*, were sequence-verified and deposited to Addgene (#'s 127374 and 127375).

Cloning of ROSA26-specific CRISPR-Cas9 construct. pX330-U6-Chimeric_BB-CBh-SPCas9 was obtained from Addgene (#42230) (Cong et al., 2013). Duplexed oligos encoding the ROSA26 intronic sgRNA sequence 5' ACTCCAGTCTTTCTAGAAGA 3' (Chu et al., 2016) were cloned into the BbsI site of this plasmid. The resultant clone, termed *pX330-sgR26*, was sequence-verified and deposited to Addgene (# 127376).

*Cloning of shRNA constructs for human RCOR2 knock down and qPCR.*_The pEN_TGmiRc3 Gateway entry vector (Shin et al., 2006) was linearized with BfuA1 and duplexed oligos containing shRNA sequences targeting RCOR2 (target mRNA sequences: sh-RCOR2_1, 5' CCGTAGGTATGGCAAAGACTT 3'; sh-RCOR2_2, 5' GATCCGCGTTGGAACCAATTA 3'; sh-RCOR2_3, 5' GGCATGCTTCTGTGGCATAAG 3'), as well as a scrambled control (target sequence: 5' CCTAAGGTTAAGTCGCCCTCG 3') were inserted. Sequence-verified clones were then input into a LR clonase reaction (Invitrogen) with the pSLIK-Neo destination vector. Sequence verification was again performed, and positive clones were co-transfected into 293T cells along with pMDLg_pRRE, pRSV-REV, and pCMV-VSVG to produce lentiviral particles. Lentivirus was concentrated using Lenti-X Concentrator (Takara). Target cells were transduced with concentrated lentivirus and TransDux (System Biosciences) to promote efficient viral entry. Transduced cells were selected with 1,000 µg/ml neomycin. Selected cells were treated with 1 µg/ml doxycycline for 48 hrs, total RNA was harvested (Qiagen) and cDNA was synthesized for qPCR reactions. Of the three above-mentioned shRCOR2 shRNA sequences, shRCOR2_1-mediated knockdown yielded the greatest RCOR2 downregulation; MCF7 cells stably expressing shRCOR2_1 were thus used in downstream qPCR assays to test hERVs, IFNs, and ISGs. Refer to Supp. Table 5 for qPCR primer pair sequences used in this analysis. qPCR reactions were run on a CFX Connect Real-Time System (Biorad), and relative fold expression was calculated using the $2^{-\Delta\Delta Ct}$ method.

Cell lines and authentication. The 4T1 and 4T1-S (Abe et al., 2016) mouse breast cancer cell lines were supplied by Dr. Ken-ichiro Seino (Hokkaido University, Sapporo, Hokkaido, Japan). Cell lines were authenticated by STR profiling (IDEXX BioResearch). Both 4T1 and 4T1-S were cultured in RPMI 1640 medium supplemented with 10% fetal bovine serum, 1% penicillin/streptomycin, 1% L-glutamine, 1 mM sodium pyruvate, 1% non-essential amino acids, and 50 μM 2-mercaptoethanol. The MC38 mouse colon adenocarcinoma cell line was supplied by Dr. Yong Lu (Wake Forest School of Medicine, Winston-Salem, NC); culture media consisted of DMEM supplemented with 10% FBS, 1% penicillin/streptomycin and 1% L-glutamine. The P815 mouse mastocytoma cell line was kindly supplied by Dr. Martha Alexander-Miller (Wake Forest School of Medicine, Winston-Salem, NC); culture medium consisted of RMPI 1640 supplemented with 10% FBS, 1% penicillin/streptomycin and 1% L-glutamine. The mouse B16-F10 melanoma cell line was attained from the Cell and Viral Vector Core Laboratory (Comprehensive Cancer Center, Wake Forest University); culture media consisted of DMEM supplemented with 10% FBS, 1% penicillin/streptomycin and 1% L-glutamine. Cell lines used for mouse studies were confirmed to be free of mycoplasma prior to implantation into mice. Cells were suspended in naked media for injections into mice.

Stable cell line generation. Expression constructs were co-transfected with pX330-sgR26 into 4T1-S cells and MC38 cells using Lipofectamine 2000 (Invitrogen), and stable cell lines termed 4T1-S-pR26, 4T1-S-pR26-mBMP7, MC38-pR26-CMVconst and MC38-pR26-CMVconst-mBMP7 were selected using either 10 µg/ml (4T1-S) or 5 µg/ml (MC38) puromycin. Genomic DNA was extracted from stable cell lines, and PCR primers (one internal to the genomically-inserted cassette and the other downstream of the 3' end of the Right ROSA26 homology arm) were used to amplify a 1135 bp fragment, confirming site-specific integration.

MHC Class I Haplotype Verification of 4T1-S Cell Line. Adherent cell cultures (4T1, 4T1-S, 4T1-S-pR26, B16-F10) were washed with PBS and dissociated with Accutase to preserve surface epitopes. Cells were resuspended in cold FACS buffer (1% FBS/PBS, 20 μm-filtered) and stained with either FITC mouse anti-mouse-H-2Kd or isotype control (BD Pharmingen #'s 553565 and 553456, respectively) for 30 minutes on ice protected from light. Cells were then washed and resuspended in FACS buffer, and analyzed on a BD Accuri C6 flow cytometer.

Western blot analysis. Stable cell lines were washed in cold PBS and then lysed in radioimmunoprecipitation assay (RIPA) buffer containing 1x protease inhibitor cocktail (Sigma #P8340) and stored at -80˚C. Upon thawing, lysates were cleared by centrifugation and protein concentration was determined by bicinchoninic acid (BCA) assay. Protein lysates were separated on a standard 5% stacking/12% resolving polyacrylamide gel, SDS-PAGE was performed, and proteins were transferred to polyvinylidene fluoride (PVDF) membranes. Membranes were blocked for 1 hr in either 5% BSA/TBST (plus 0.5% Tween-20) for anti-BMP7 staining, or 5% nonfat dry milk/TBST (plus 0.5% Tween-20) for anti-βactin staining. Primary antibodies were incubated overnight at 4˚C in BSA/TBST or milk/TBST (for anti-BMP7 or anti-βactin staining, respectively). Blots were washed 3 x 10 min with TBST, secondary antibodies were added in milk/TBST and incubated for 1 hr at room temperature, blots were again washed, and WesternBright ECL was used for developing blots (Advansta #K12045-D20). Antibodies used for western blot were: rabbit-anti-BMP7 (ab27569, 1:2000), goat-anti-rabbit IgG-HRP (Santa Cruz Biotechnology, #sc-2004, 1:5000), mouse-anti-βactin (Sigma A5441, 1:10000), and goat-anti-mouse IgG-HRP (Santa Cruz Biotechnology, #sc-2005, 1:10000).

Immunofluorescence staining of tumor-infiltrating CTLs. A portion of tumor was fixed in 4% paraformaldehyde for 2 hrs on ice, washed in PBS, equilibrated in 30% sucrose (w/v) overnight at 4˚C, then OCT-embedded and stored at -80˚C. Ten-micron sections were cut by cryostat, dried and rehydrated in PBS. Slides were incubated in permeabilization buffer (0.1% Tween-20, 0.01% Triton-X 100 in PBS) for 20 min at RT, then blocked (4% BSA, 4% normal rat serum) for 30 min at RT. Primary anti-CD8a antibody mixture (1:50 anti-CD8a, ThermoFisher Scientific #MCD-0804; diluted with 2% BSA and 0.08% normal rat serum in PBS) was then added and sections were incubated overnight at 4˚C. Washed sections were counterstained with 5 µg/ml 4',6-diamidino-2-phenylindole (DAPI), washed again, and then mounted with ProLong Gold AntiFade (ThermoFisher Scientific). Immunofluorescent images were obtained for 5-6 random fields per tumor section (40x magnification) using the Mantra Quantitative Pathology Workstation (Perkin Elmer). Total numbers of CD8a+ CTLs per field were quantitated by manual counting.

Tumor dissociation and flow cytometry analysis of tumor-infiltrating CTLs. Portions of tumors (MC38 model) at the experimental endpoint were diced and digested with 1 mg/ml collagenase IV (Worthington) for 2 hrs at 37˚C. The tumor digestion was passed through a 70 µm cell strainer, resuspended in 5 ml RPMI-1640 medium, underlaid with 4 ml of histopaque-1083 (Sigma-Aldrich), and spun in a swinging-bucket tabletop centrifuge at 400 x g for 30 min without braking. The interface containing TILs was harvested and washed in RPMI, then resuspended in RPMI and counted (Note: this tumor dissociation protocol was the same for harvesting cells for single cell RNAseq analysis). Cells were stained with LIVE/DEAD Fixable Violet Dead Cell Stain Kit (ThermoFisher Scientific) for 30 min on ice (protected from light, as well as subsequent steps). Non-specific staining was blocked by incubating cells with TruStain FcX anti-mouse CD16/32 antibody (Biolegend #101319) in FACS buffer (1% FBS in PBS, 20-µm-filtered) for 5 min. Fluorescently-conjugated antibodies (1:20 anti-CD8a-BV510, 1:40 anti-CD3-PE, and 1:20 anti-CD45-BV785; Biolegend #100752, 100206, and 103149, respectively) were added and cells were stained on ice for 30 min. Cells were washed, resuspended in FACS buffer, and analyzed using a BD LSRFortessa X-20 Analyzer (BD Biosciences). FCS files were analyzed by FCS Express 4 Flow Research Edition (De Novo Software).

Supplementary References

Abe, H., Wada, H., Baghdadi, M., Nakanishi, S., Usui, Y., Tsuchikawa, T., et al. (2016). Identification of a highly immunogenic mouse breast cancer sub cell line, 4T1-S. *Hum Cell*. doi: 10.1007/s13577-015-0127-1.

Ayers, M., Lunceford, J., Nebozhyn, M., Murphy, E., Loboda, A., Kaufman, D.R., et al. (2017). IFN-gamma-related mRNA profile predicts clinical response to PD-1 blockade. *J Clin Invest* 127(8)**,** 2930-2940. doi: 10.1172/jci91190.

Benita, Y., Cao, Z., Giallourakis, C., Li, C., Gardet, A., and Xavier, R.J. (2010). Gene enrichment profiles reveal T-cell development, differentiation, and lineage-specific transcription factors including ZBTB25 as a novel NF-AT repressor. *Blood* 115(26)**,** 5376-5384. doi: 10.1182/blood-2010-01-263855.

Bertucci, F., Finetti, P., Simeone, I., Hendrickx, W., Wang, E., Marincola, F.M., et al. (2018). The immunologic constant of rejection classification refines the prognostic value of

conventional prognostic signatures in breast cancer. *Br J Cancer* 119(11), 1383-1391. doi: 10.1038/s41416-018-0309-1.
Bindea, G., Mlecnik, B., Tosolini, M., Kirilovsky, A., Waldner, M., Obenauf, A.C., et al. (2013). Spatiotemporal dynamics of intratumoral immune cells reveal the immune landscape in human cancer. *Immunity* 39(4), 782-795. doi: 10.1016/j.immuni.2013.10.003.
Chifman, J., Pullikuth, A., Chou, J.W., Bedognetti, D., and Miller, L.D. (2016). Conservation of immune gene signatures in solid tumors and prognostic implications. *BMC Cancer* 16(1), 911. doi: 10.1186/s12885-016-2948-z

10.1186/s12885-016-2948-z [pii].
Chu, V.T., Weber, T., Graf, R., Sommermann, T., Petsch, K., Sack, U., et al. (2016). Efficient generation of Rosa26 knock-in mice using CRISPR/Cas9 in C57BL/6 zygotes. *BMC Biotechnol* 16, 4. doi: 10.1186/s12896-016-0234-4.
Cong, L., Ran, F.A., Cox, D., Lin, S., Barretto, R., Habib, N., et al. (2013). Multiplex genome engineering using CRISPR/Cas systems. *Science* 339(6121), 819-823. doi: 10.1126/science.1231143.
Fehrenbacher, L., Spira, A., Ballinger, M., Kowanetz, M., Vansteenkiste, J., Mazieres, J., et al. (2016). Atezolizumab versus docetaxel for patients with previously treated non-small-cell lung cancer (POPLAR): a multicentre, open-label, phase 2 randomised controlled trial. *Lancet* 387(10030), 1837-1846. doi: 10.1016/s0140-6736(16)00587-0.
Liu, J., Lichtenberg, T., Hoadley, K.A., Poisson, L.M., Lazar, A.J., Cherniack, A.D., et al. (2018). An Integrated TCGA Pan-Cancer Clinical Data Resource to Drive High-Quality Survival Outcome Analytics. *Cell* 173(2), 400-416.e411. doi: 10.1016/j.cell.2018.02.052.
Max Gordon, T.L. (2017). "forestplot: Advanced Forest Plot Using 'grid' Graphics". version 1.7.2 ed.).
Rooney, M.S., Shukla, S.A., Wu, C.J., Getz, G., and Hacohen, N. (2015). Molecular and genetic properties of tumors associated with local immune cytolytic activity. *Cell* 160(1-2), 48-61. doi: 10.1016/j.cell.2014.12.033.
Saltz, J., Gupta, R., Hou, L., Kurc, T., Singh, P., Nguyen, V., et al. (2018). Spatial Organization and Molecular Correlation of Tumor-Infiltrating Lymphocytes Using Deep Learning on Pathology Images. *Cell Rep* 23(1), 181-193.e187. doi: 10.1016/j.celrep.2018.03.086.
Shin, K.J., Wall, E.A., Zavzavadjian, J.R., Santat, L.A., Liu, J., Hwang, J.I., et al. (2006). A single lentiviral vector platform for microRNA-based conditional RNA interference and coordinated transgene expression. *Proc Natl Acad Sci U S A* 103(37), 13759-13764. doi: 10.1073/pnas.0606179103.
Therneau, T.M. (2015). "A Package for Survival Analysis in S". version 2.38 ed.).
Thorsson, V., Gibbs, D.L., Brown, S.D., Wolf, D., Bortone, D.S., Ou Yang, T.H., et al. (2018). The Immune Landscape of Cancer. *Immunity* 48(4), 812-830.e814. doi: 10.1016/j.immuni.2018.03.023.
